# Recent progress in chemistry and bioactivity of monoterpenoid indole alkaloids from the genus *gelsemium*: a comprehensive review

**DOI:** 10.1080/14756366.2022.2155639

**Published:** 2023-01-11

**Authors:** Lin Wang, Siyu Chen, Xun Gao, Xiao Liang, Weichen Lv, Dongfang Zhang, Xin Jin

**Affiliations:** a School of Pharmacy, China Medical University, Shenyang, China; b China Medical University-Queen’s University of Belfast Joint College, China Medical University, Shenyang, China; c Jiangsu Institute Marine Resources Development, Jiangsu Ocean University, Lianyungang, China; d School of Pharmacy, Liaoning University, Shenyang, China; e Department of Clinical Medicine, Dalian University, Dalian, China

**Keywords:** Gelsemium genus, monoterpenoid indole alkaloids, new chemical structure, synthetic chemistry, biological activity

## Abstract

Monoterpenoid indole alkaloids (MIAs) represent a major class of active ingredients from the plants of the genus *Gelsemium*. Gelsemium MIAs with diverse chemical structures can be divided into six categories: gelsedine-, gelsemine-, humantenine-, koumine-, sarpagine- and yohimbane-type. Additionally, gelsemium MIAs exert a wide range of bioactivities, including anti-tumour, immunosuppression, anti-anxiety, analgesia, and so on. Owing to their fascinating structures and potent pharmaceutical properties, these gelsemium MIAs arouse significant organic chemists’ interest to design state-of-the-art synthetic strategies for their total synthesis. In this review, we comprehensively summarised recently reported novel gelsemium MIAs, potential pharmacological activities of some active molecules, and total synthetic strategies covering the period from 2013 to 2022. It is expected that this study may open the window to timely illuminate and guide further study and development of gelsemium MIAs and their derivatives in clinical practice.

## Graphical abstract

**Figure:**
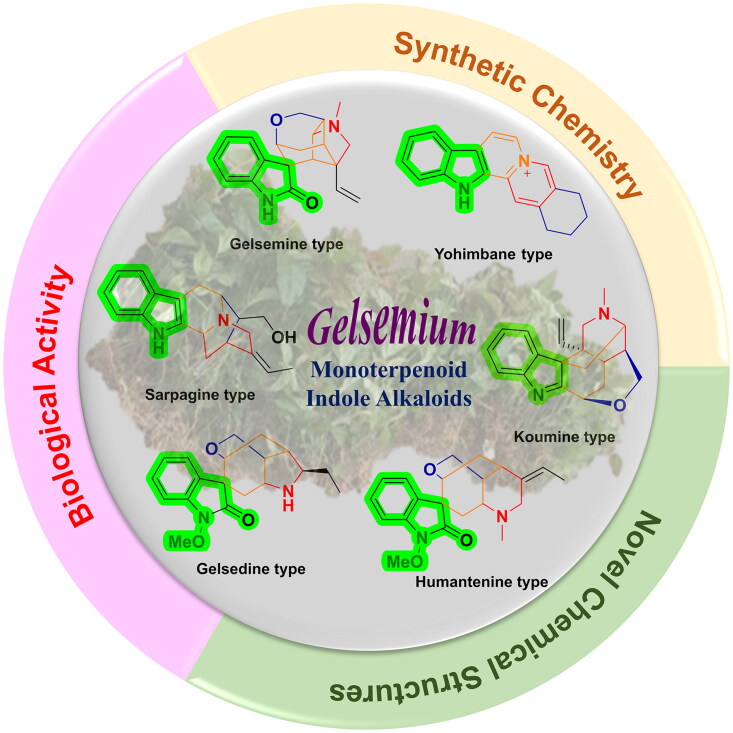

## Introduction

Derivatives of monoterpenoid indole alkaloids (MIAs) represent a class of active secondary metabolites mostly isolated from the plants of *the Gelsemium* genus (Loganiaceae), mainly in three known *Gelsemium* species, including *G. sempervirens*, *G. elegans* and *G. rankinii*^1^. These plants are mainly distributed in southern China, Asia, and North America, and have been broadly used as a folk herbal medicine for clinical treatment for hundreds of years^2^. MIAs are particularly concentrated in the roots of *Gelsemium* plants, accounting for the content of approximately 0.5%, whereas the stems, fruits, branches, and leaves also contain smaller amounts^3^. Since the discovery of the first MIA in 1959, more than 100 kinds of representative MIAs with complex structures have been widely extracted from these *Gelsemium* plants^4^. The majority of gelsemium MIAs have been found to exhibit a plethora of notable pharmacological properties, especially anti-tumour, immunosuppressive, anxiolytic, and analgesic characteristics, reflecting their great potential as lead compounds in new drug development^5–8^.

**Figure 1.**
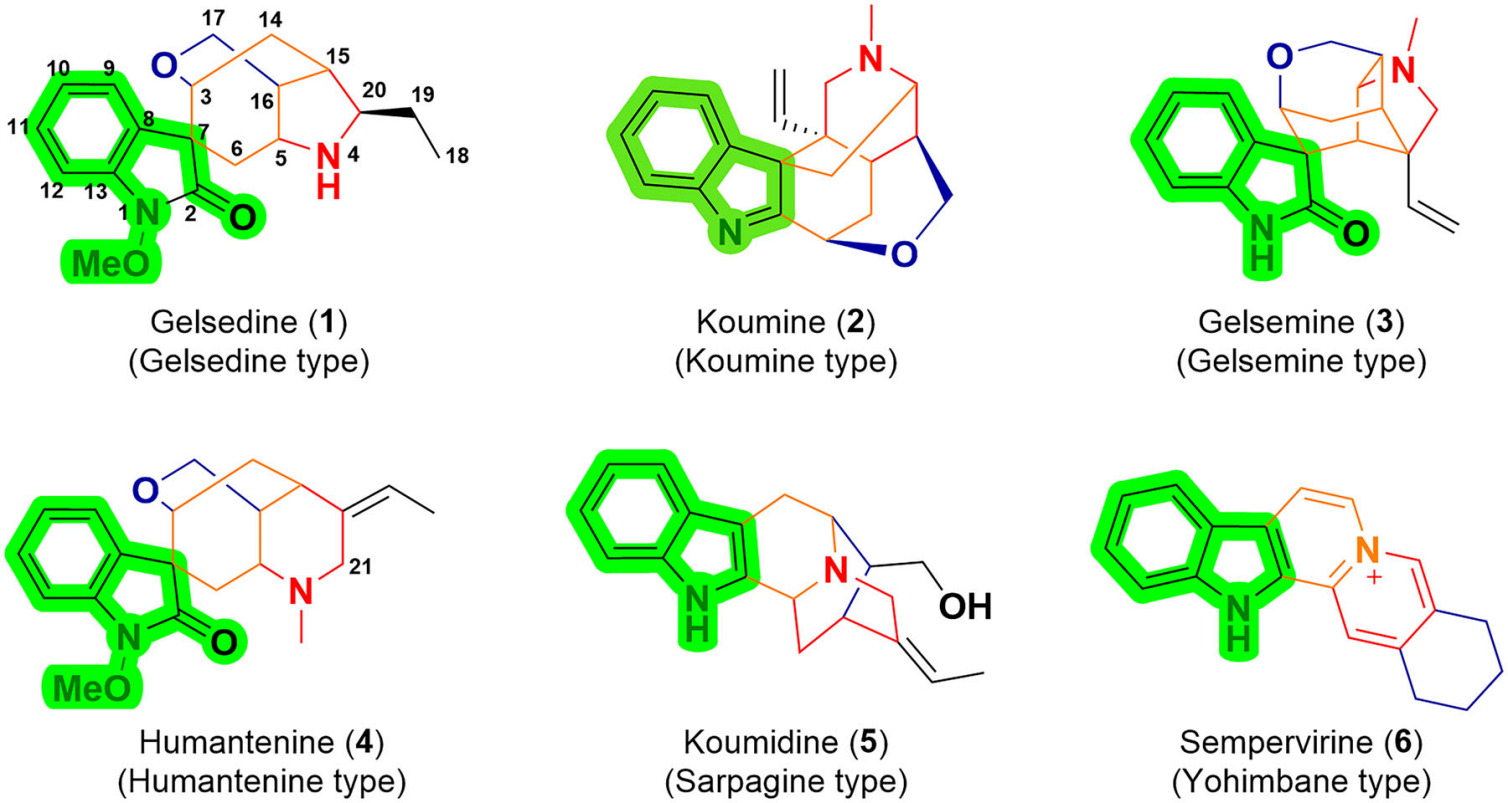
Six representative members of gelsemium MIAs. Gelsemium MIAs can be classified into six categories: gelsedine-, koumine-, gelsemine-, humantenine-, sarpagine-, and yohimbane-type. Gelsemine-type, humantenine-type and gelsedine-type alkaloids bear peculiar *spiro*-indolinone nuclei, while koumine-, sarpagine- and yohimbane-type alkaloids have normal indole groups.

In terms of structure type, gelsemium MIAs possess characteristic chemical structures containing polycyclic monoterpene portions and indole, oxindole, or bisindole nuclei^9^. Gelsemium MIAs can be classified into six categories: gelsedine-, gelsemine-, humantenine-, koumine-, sarpagine- and yohimbane-type, on the basis of their structural features^10^ (Figure 1). Among them, gelsemine-type, humantenine-type and gelsedine-type alkaloids bear peculiar *spiro*-indolinone nuclei, while koumine-, sarpagine- and yohimbane-type alkaloids have normal indole groups. The structural skeletons of their monoterpene parts incorporate sterically compact and dense polycyclic architectures and multiple stereocenters, forming privileged chemical diversity and structural complexity of gelsemium MIAs. These exceptional structural properties of gelsemium MIAs render them sophisticated challenges for total synthesis and structure modification and attracted considerable attention from synthetic scientists. Historically, a vast number of total synthetic works on gelsemium MIAs have been reported^11–13^.

Figure 2.The chemical structures of novel gelsedine-type alkaloids.

**Figure:**
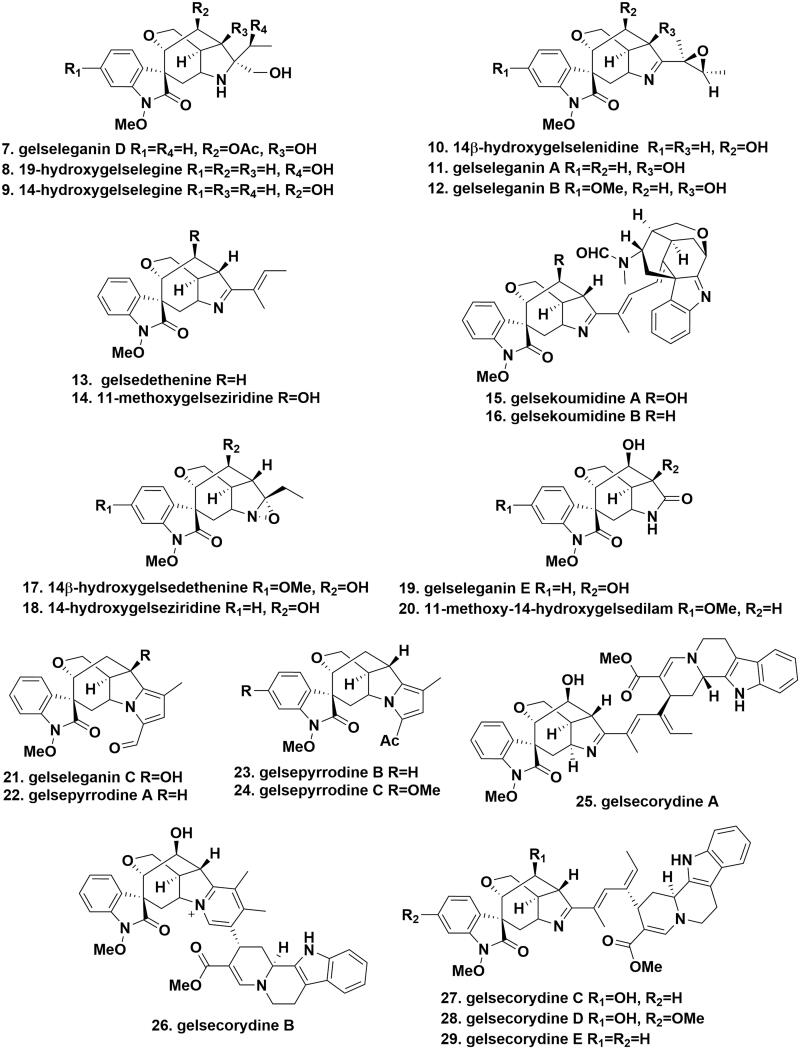

**Figure:**
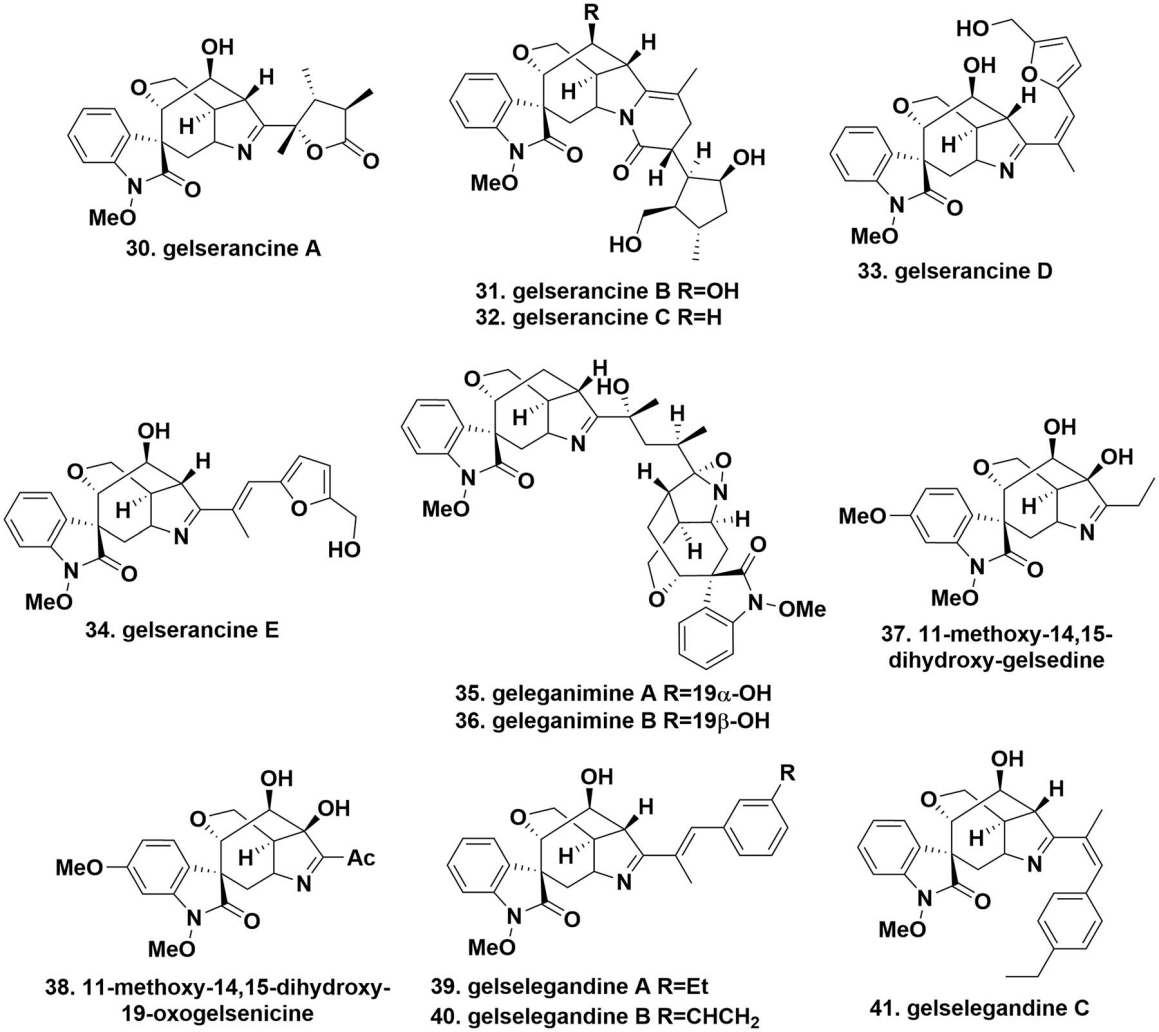

Jin’s group (2014) previously reviewed the phytochemistry, pharmacology, and toxicology together with their traditional use of the genus *Gelsemium*, whereas Carter’s group (2019) described the synthetic strategies towards the gelsemine- and gelsedine-type MIAs between 2005 and 2016^14^^,^^15^. However, these reviews did not provide a comprehensive review of all types of gelsemium MIAs, especially in aspects of their frontier pharmacological effects as well as chemical structures and syntheses. A large number of breakthroughs in their novel compounds, biological activities, and more elegant total syntheses have been reported over the past decade. As such, this review is intended to comprehensively summarise the representative examples covering from 2013 to 2022 with the following novel objectives: (1) a comprehensive presentation of novel gelsemium MIAs and more elegant total synthetic methodologies; (2) a focus on recent their bioactivities of gelsemium MIAs, mainly involving specific biotarget and mechanism of actions; (3) an overview of advice how gelsemium MIAs can be utilised as promising candidates in further studies. Finally, we hope that this review will provide an insight into rational study and development of gelsemium MIAs and their derivatives in further clinical practice.

## Novel chemical structures of gelsemium MIAs

Since 2013, a total of 70 novel MIAs have been isolated from the *Gelsemium* genus, mainly *G. elegans*. The structural types of gelsemium MIAs are mainly focussed on gelsedine-type, humantenine-type, and koumine-type. These gelsemium MIAs groups will be discussed in the following paragraphs (Table 1).

**Table 1.** Summary of novel MIAs from the genus of *Gelsemium.*

Structure types	Compounds (Number)	Parts of plants	Isolation sources	Structure identification	Reference
Gelsedine-type alkaloids	gelseleganins D (**7**), A (**11**), B (**12**), E (**19**), and C (**21**)	Leaves and branches	*G. elegans*	UV, HR-ESI-MS, ^13^C and ^1^H NMR	^16^
	19-hydroxygelselegine (**8**)	Stems	*G. elegans*	HR-ESI-MS, ECD, ^13^C and ^1^H NMR, iJ/dJ-DP4 analysis	^17^
	14-hydroxygelselegine (**9**)	Roots	*G. elegans*	HR-ESI-MS, ECD, ^13^C and ^1^H NMR, X-ray analysis	^15^
	14β-hydroxygelselenidine (**10**), 11-methoxygelseziridine (**14**), and 14*β*-hydroxygelsedethenine (**17**)	Aerial parts	*G. elegans*	UV, IR, HR-ESI-MS, ECD, ^13^C and ^1^H NMR	^18^
	gelsedethenine (**13**)	Roots	*G. elegans*	HR-ESI-MS, ECD, ^13^C and ^1^H NMR, X-ray analysis	^19^
	gelsekoumidines A (**15**) and B (**16**)	Roots	*G. elegans*	HR-ESI-MS, ECD, ^13^C and ^1^H NMR, X-ray analysis	^20^
	14-hydroxygelseziridine (**18**)	Roots and stems	*G. elegans*	HR-ESI-MS, ECD, ^13^C and ^1^H NMR	^21^
	11-methoxy-14-hydroxygelsedilam (**20**), 11-methoxy-14,15-dihydroxygelsedine (**37**), and 11-methoxy-14,15-dihydroxy-19-oxogelsenicine (**38**)	Leaves and branches	*G. elegans*	HR-FAB-MS, ^13^C and ^1^H NMR	^22^
	gelsepyrrodines A (**22**), B (**23**), and C (**24**)	Roots	*G. elegans*	HR-ESI-MS, ECD, ^13^C and ^1^H NMR	^23^
	gelsecorydines A (**25**), B (**26**), C (**27**), D (**28**), and E (**29**)	Fruits	*G. elegans*	UV, IR, HR-ESI-MS, ECD, ^13^C and ^1^H NMR	^24^
	gelserancines A (**30**), B (**31**), C (**32**), D (**33**), and E (**34**)	Roots	*G. elegans*	HR-ESI-MS, ECD, ^13^C and ^1^H NMR	^25^
	geleganimines A (**35**) and B (**36**)	Aerial parts	*G. elegans*	HR-ESI-MS, ECD, ^13^C and ^1^H NMR	^26^
	gelselegandines A (**39**), B (**40**), and C (**41**)	Roots	*G. elegans*	HR-ESI-MS, ECD, ^13^C and ^1^H NMR	^27^
Humantenine-type alkaloids	19,20-epoxyhumantenine (**42**)	Roots	*G. elegans*	HR-ESI-MS, ECD, ^13^C and ^1^H NMR, X-ray analysis	^19^
	gelselegandines D (**43**) and E (**44**)	Roots and stems	*G. elegans*	HR-ESI-MS, ECD, ^13^C and ^1^H NMR	^28^
	geleganidines A (**45**) and C (**46**)	Roots	*G. elegans*	HR-ESI-MS, ECD, ^13^C and ^1^H NMR, X-ray analysis	^29^
	11-hydroxyhumantenine *N*_4_-oxide (**47**), *N*-desmethoxyhumantenine *N*_4_-oxide (**48**), and gelstriamine A (**50**)	Stems	*G. elegans*	HR-ESI-MS, ECD, ^13^C and ^1^H NMR, iJ/dJ-DP4 analysis	^17^
	*N*_4_-methyl-19,20-dihydrorankinidine (**49**)	Roots	*G. elegans*	HR-ESI-MS, ECD, ^13^C and ^1^H NMR, X-ray analysis	^15^
	14β,20α-dihydroxydihydrorankinidine (**51**), 11-methoxy-19,20α-dihydroxydihydrorankinidin (**52**), and norhumantenine A (**53**)	Leaves and stems	*G. elegans*	HR-ESI-MS, ^13^C and ^1^H NMR	^30^
Koumine-type alkaloids					
	19-dehydrokouminol (**54**), koureamine (**55**), (*4R*)-dihydrokoumine *N*_4_-oxide (**56**), (4*S*)-dihydrokoumine *N*_4_-oxide (**57**), isodihydrokoumine-*N*_1_-oxide (**65**), and (*4R*)-isodihydrokoumine-*N*_4_-oxide (**66**)	Roots	*G. elegans*	HR-ESI-MS, ECD, ^13^C and ^1^H NMR, X-ray analysis	^31^
	*N*_4_-demethyl-21-dehydrokoumine (**58**), 21α-hydroxylkoumine (**59**), 21β-hydroxylkoumine (**60**), and (*19S*)-hydroxydihydrokoumine *N*_4_-oxide (**61**)	Leaves and stems	*G. elegans*	UV, HR-ESI-MS, ECD, ^13^C and ^1^H NMR	^30^
	21-oxokoumine (**62**) and furanokoumine (**63**)	Roots	*G. elegans*	UV, HR-ESI-MS, ECD, ^13^C and ^1^H NMR	^32^
	18, 19-dihydro-21-oxokoumine (**64**)	Roots	*G. elegans*	HR-ESI-MS, ECD, ^13^C and ^1^H NMR, X-ray analysis	^15^
Yohimbane-type alkaloids	sempervirinoxide (**67**) and *seco*-semperviroic acid (**68**)	Leaves and stems	*G. elegans*	UV, HR-ESI-MS, ECD, ^13^C and ^1^H NMR	^30^
	gelsechizines A (**69**) and B (**70**)	Fruits	*G. elegans*	UV, IR, HR-ESI-MS, ECD, ^13^C and ^1^H NMR	^33^
Sarpagine-type alkaloids	*epi*-koumidine *N*_4_-oxide (**71**)	Stems	*G. elegans*	HR-ESI-MS, ECD, ^13^C and ^1^H NMR, iJ/dJ-DP4 analysis	^17^
Gelsemine-type alkaloids	(*4R*)-19-oxo-gelsevirine *N*_4_-oxide (**72**)	Roots	*G. elegans*	HR-ESI-MS, ECD, ^13^C and ^1^H NMR, X-ray analysis	^19^
Other-type alkaloids	10,11-dimethoxy-*N*_1_-demethoxy-gelsemamide (**73**) and 11-demethoxy-gelsemazonamide (**74**)	Roots	*G. elegans*	HR-ESI-MS, ECD, ^13^C and ^1^H NMR, X-ray analysis	^19^
	geleganamide (**75**)	Aerial parts	*G. elegans*	HR-ESI-MS, ECD, ^13^C and ^1^H NMR	^26^
	geleganidine B (**76**)	Roots	*G. elegans*	HR-ESI-MS, ECD, ^13^C and ^1^H NMR, X-ray analysis	^29^

ECD: electronic circular dichroism; ESI: electron spray ionisation; FAB: fast atom bombardment; HR-MS: high resolution mass spectrometry; IR: infra-red; NMR: nuclear magnetic resonance; UV: ultraviolet.

### Gelsedine-type alkaloids

**Figure 3.**
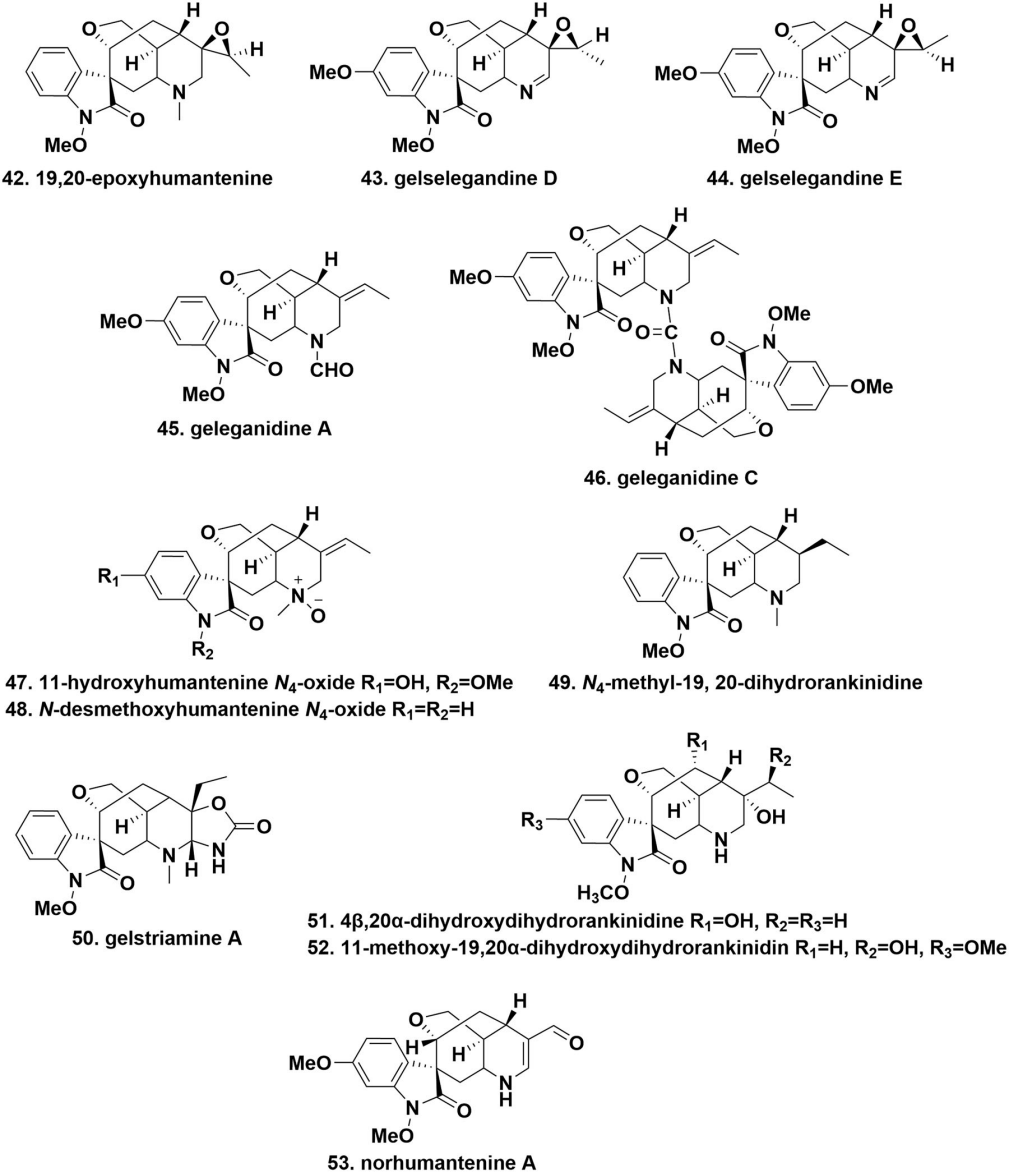
The chemical structures of novel gelsedine-type alkaloids.

Gelsedine**-**type alkaloids occupy the largest portion of the gelsemium MIAs, whose intricate frameworks share a characteristic *spiro*-*N*-methoxyindoleone chromophore, an oxabicyclo[3.2.2]nonane ring system, and a versatile functionalised pyrrolidine ring inserted in their complex cage-like skeletons. The main differences among individual gelsedine-type members are different substituents at C11, 14, 15, and 20. Gelseleganin D (**7**), 19-hydroxygelselegine (**8**), and 14-hydroxygelselegine (**9**), belonging to classical gelsedine-type skeleton containing a methylol group at C20, were isolated from the different parts of *G. elegans*^16–18^. 14β-Hydroxygelselenidine (**10**), gelseleganins A (**11**), and B (**12**) were obtained from the aerial parts of *G. elegans*. These compounds represented a rare class of MIAs carrying a 2,3-epoxybutane moiety at C20^17^^,^^19^. Gelsedethenine (**13**) and 11-methoxygelseziridine (**14**) were isolated from the roots and aerial parts of *G. elegans*, respectively, which structurally featured a particular *trans*-butenyl group at C20^19^^,^^20^. Gelsekoumidines A (**15**) and B (**16**) were two pairs of atropoisomeric bisindole alkaloids from the roots of *G. elegans*. Gelsekoumidines A and B represented an unprecedented class of *seco*-koumine-gelsedine-type alkaloids containing a unique 20,21-*seco*-koumine skeleton fused with a gelsedine scaffold via a double bond bridge. The only difference between these two compounds was that gelsekoumidine A had a hydroxyl group at C14^21^. 14β-Hydroxygelsedethenine (**17**) and 14-hydroxygelseziridine (**18**) were obtained from the aerial parts of *G. elegans.* Structural comparison with gelsedine (**1**) displayed that the two compounds had an α-configuration of the 1,2-oxaziridine group located between C20 and N4^19^,^22^. Gelseleganin E (**19**) and 11-methoxy-14-hydroxygelsedilam (**20**) were isolated from the leaves and branches of *G. elegans*, which possessed a particular lactam ring^17^^,^^23^. Gelseleganin C (**21**) and gelsepyrrodines A (**22**), B (**23**), and C (**24**) were isolated from the leaves, branches, and roots of *G. elegans*, in which a pyrrole ring was incorporated into their gelsedine-type skeleton. The main difference was the replacement of an additional aldehyde in the pyrrole ring in the former two by an acetyl group in the latter two^17^^,^^24^. Gelsecorydines A (**25**), B (**26**), C (**27**), D (**28**), and E (**29**), five bisindole alkaloids with novel chemical skeleton, were obtained from the fruits of *G. elegans*. Their heterodimeric framework incorporated a gelsedine-type alkaloid and a modified corynanthe-type monomer. Especially, gelsecorydine B possessed an unprecedented caged structure with a 6/5/7/6/5/6 heterohexacyclic ring system via a direct pyridine ring linkage^25^. Gelserancines A (**30**), B (**31**), C (**32**), D (**33**), and E (**34**), five unusual gelsedine-type derivatives, were separated from the roots of *G. elegans*. The structure of gelserancine A incorporated a rare trimethyl-dihydrofuranone building block at C20. In gelserancines B and C, their gelsedine-type frameworks are bound with an additional pyridine ring with a 5-hydroxy-2-(hydroxymethyl)-3-methylcyclopentyl moiety at N4 and C19. Gelserancines D and E were a pair of E/Z tautomer, in which the 14-hydroxygelsenicine unit was connected to a 2-hydroxymethyl furan ring via a C19-C1' conjugated bridge^26^. Geleganimines A (**35**) and B (**36**), two trace nonsymmetric bisindole alkaloids, were isolated from the aerial parts of *G. elegans*. Geleganimines A and B belonged to two epimers consisting of gelsenicine and gelseziridine moieties via a 3-carbon alkanoic chain^27^. 11-Methoxy-14,15-dihydroxygelsedine (**37**) and 11-methoxy-14,15-dihydroxy-19-oxogelsenicine (**38**) were got from the ethanol extracts of the leaves and branches of *G. elegans*. The difference between them was that the ethyl moiety in compound 37 was replaced by an acetyl group in compound 38^23^. Gelselegandines A (**39**), B (40), and C (4**1**), isolated from the roots of *G. elegans*, possess an unprecedented gelsedine-type core structure incorporating an additional C_9_ aromatic unit as a side chain. Gelselegandines A and C belonged to a pair of *cis*- and *trans*-isomers, whereas gelselegandine B existed in the replacement of the ethyl group by a vinyl moiety that was not in accordance with the two formers^28^ (Figure 3).

### Humantenine-type alkaloids

**Figure 4.**
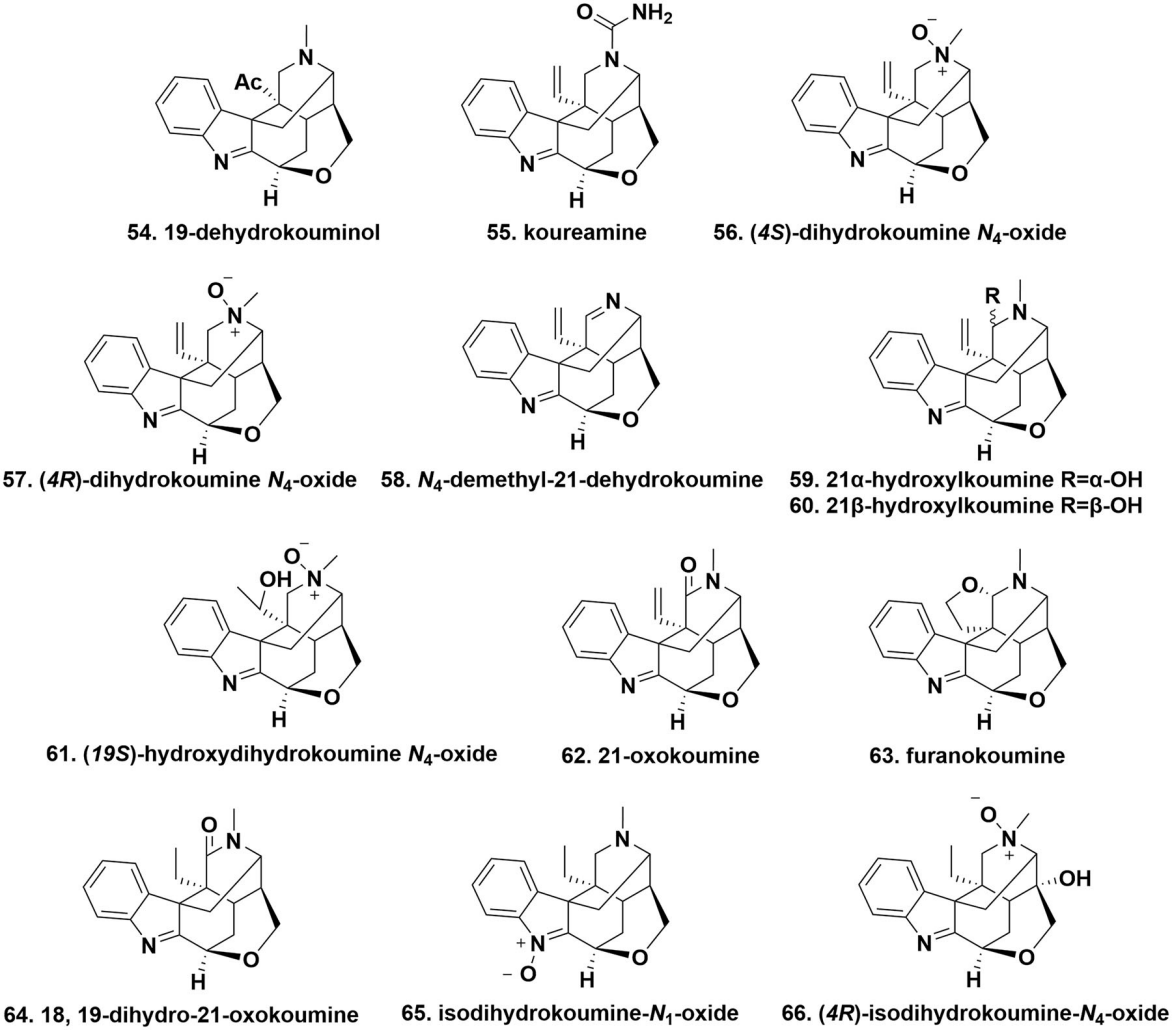
The chemical structures of novel humantenine-type alkaloids.

The chemical structures of humantenine-type alkaloids are quite similar to those of gelsemine-type alkaloids, having an oxindole group, but adding a C21 carbon and a C19-C20 double bond. 19,20-Epoxyhumantenine (**42**), gelselegandines D (**43**), and E (**44**), with a rare epoxypropyl ring at C19 and 20, were isolated from the roots and stems of *G. elegans*. The difference was that the configuration of C19 near the epoxypropyl ring was *S* and *R* in gelselegandines D and E, respectively^20^^,^^29^. Geleganidines A (**45**) and C (**46**), two unusual humantenine-type alkaloids, were isolated from the roots of *G. elegans*. Particularly, geleganidine A carried a formamide moiety at N4 in its molecular structure. Geleganidine C was a novel dimer of geleganidine A connected by a carbonyl group to form a rare urea-containing substructure^30^. 11-Hydroxyhumantenine *N*_4_-oxide (**47**) and *N-*desmethoxyhumantenine *N*_4_-oxide (**48**) were isolated from the stems of *G. elegans*. Both of them owned a N-O coordinate linkage at N4, however, the distinct difference was the methoxy group and hydroxy substitution at C11 and N1 in compound 47^18^. *N*_4_-methyl-19,20-dihydrorankinidine (**49**) and gelstriamine A (**50**) were isolated from the roots and stems of *G. elegans*, respectively. In compound 49, the C20 olefin moiety was changed to an ethyl group, compared with humantenine. As mimics of compound 49, gelstriamine A featured a unique hexahydrooxazolo[4,5-b]pyridin-2(3*H*)-one moiety at C20 and C21, forming an abnormal 6/5/7/6/6/5 heterohexacyclic core^16^^,^^18^. 14β,20α-Dihydroxydihydrorankinidine (**51**), 11-methoxy-19,20α-dihydroxydihydrorankinidin (**52**), and norhumantenine A (**53**) were purified from the leaves and vine stems of *G. elegans*. The structure of compounds 51 and 52 was similar to that of rankinidine, except for the reduction of the C19-C20 double bond with a location of a hydroxy group at C20. A comparison of structural differences showed the replacement of the C18/C21 subunits by those from an α,β-unsaturated formyl functionality in norhumantenine A^31^ (Figure 4).

### Koumine-type alkaloids

**Figure 5.**
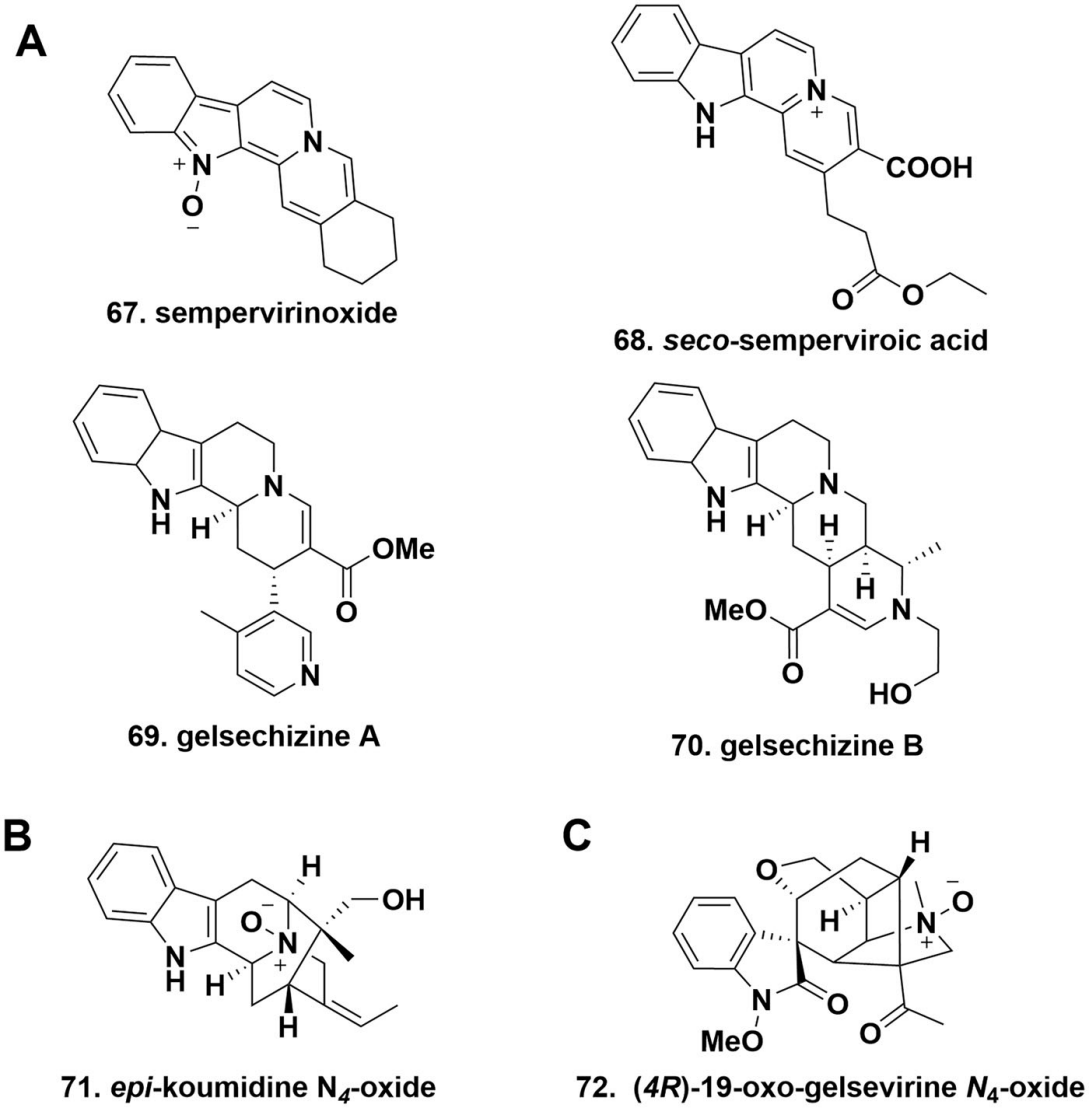
The chemical structures of novel koumine-type alkaloids.

Koumine-type alkaloids own an indole-fused cage-shaped scaffold having a terminal vinyl group that are biogenetically derived from sarpagine-type alkaloids. 19-Dehydrokouminol (**54**), koureamine (**55**), (*4 R*)-dihydrokoumine *N*_4_-oxide (**56**), (*4S*)-dihydrokoumine *N*_4_-oxide (**57**) were extracted from the roots of *G. elegans*. The terminal vinyl group at C20 in koumine (**2**) was substituted by an acetyl group in 19-dehydrokouminol. Koureamine represented the first koumine-type alkaloid with a urea group. (*4 R*)-Dihydrokoumine *N*_4_-oxide and (*4S*)-dihydrokoumine *N*_4_-oxide belonged to a pair of enantiomers of *N_4_*-oxide derivatives^32^. *N*_4_-demethyl-21-dehydrokoumine (**58**), 21α-hydroxylkoumine (**59**), 21β-hydroxylkoumine (**60**), and (*19S*)-hydroxydihydrokoumine *N*_4_-oxide (**61**) were isolated from the leaves and vine stems of *G. elegans. N*_4_-Demethyl-21-dehydrokoumine represented the first koumine-type alkaloid without a N4-methyl group. Compound 61 comprised an α-hydroxylethyl group in place of the terminal vinyl group in compound 57^31^. 21-Oxokoumine (6**2**) and furanokoumine (**63**), two new gelsemium MIAs, were isolated from the roots of *G. elegans*. As compared with koumine, additional carbonyl oxygen was attached to C21 in 21-oxokoumine and a tetrahydrofuran ring is located at C20 and C21 in furanokoumine^33^. 18, 19-Dihydro-21-oxokoumine (**64**), isodihydrokoumine-*N*_1_-oxide (**65**), and (*4 R*)-isodihydrokoumine-*N*_4_-oxide (**66**) were isolated from the roots of *G. elegans*. Structurally, these compounds differed from the prototype of koumine-type alkaloids existing in an ethyl group instead of the terminal vinyl group. Compound 64 embodied a carbonyl substitution at C21 that was similar to 21-oxokoumine. Compounds 65 and 66 belonged to N-oxide derivatives. More in detail, the *N*_1_-O dative covalent bond in the former was switched to *N*_4_-O one in the latter^16^^,^^32^ (Figure 5).

### Yohimbane-type alkaloids

**Figure 6.**
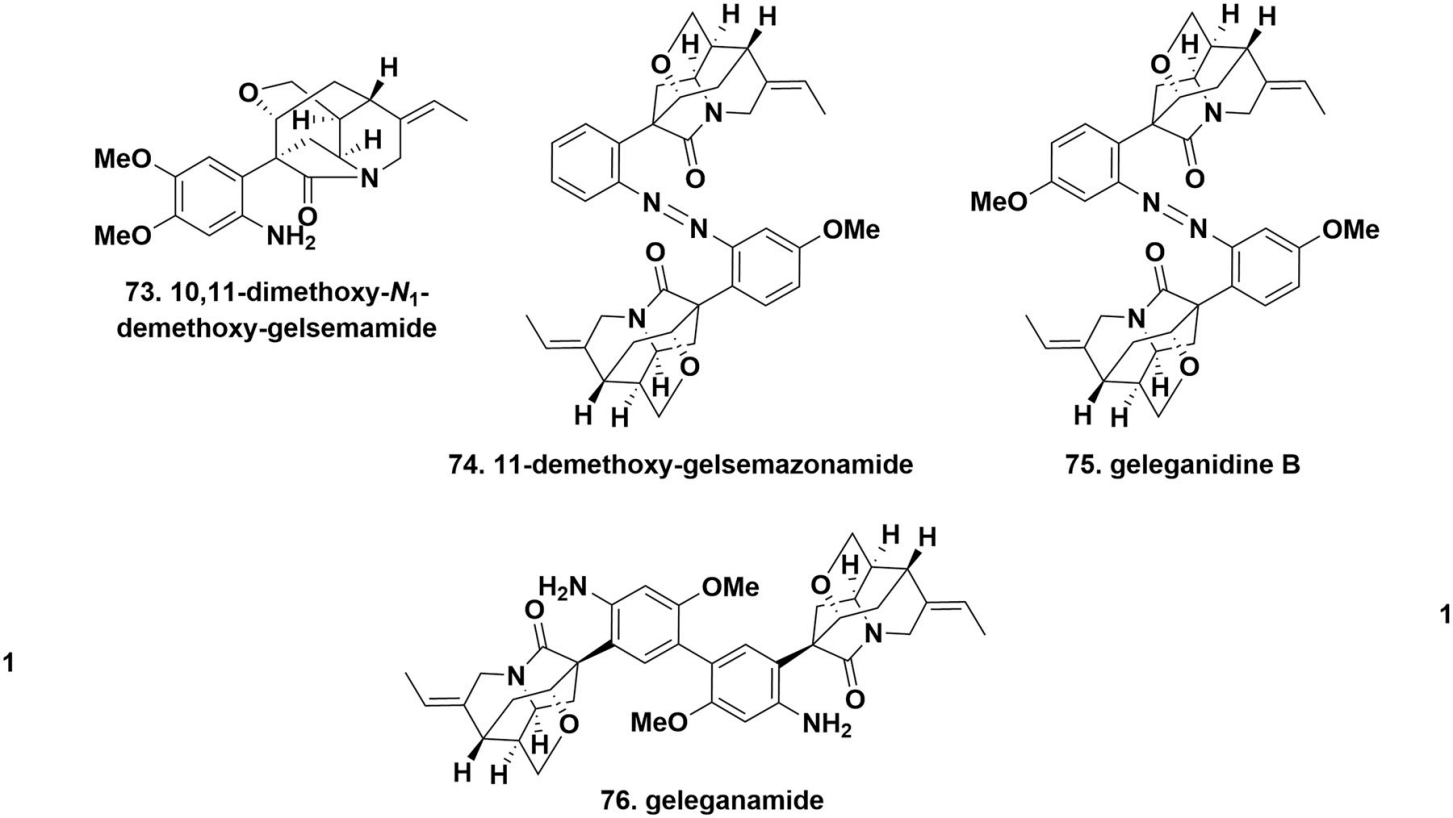
The chemical structures of novel gelsemium MIAs alkaloids. (A). yohimbane-type; (B). sarpagine-type; (C). gelsemine-type.

In the core structure of yohimbane-type alkaloids, the indole unit is adjacent to a 7,8,9,10-tetrahydropyrido[1,2-*b*]isoquinolin-5-ium. Sempervirinoxide (**67**) and *seco*-semperviroic acid (**68**), two novel MIAs, were isolated from the leaves and vine stems of *G. elegans*. Sempervirinoxide possessed an additional O atom located at N1 to form a N-O dative covalent bond. In particular, the E ring in *seco*-semperviroic acid was opened in 9/9a. Sempervirinoxide and *seco*-semperviroic acid represented the first N_1_-oxide and the first *seco*-E-ring yohimbane-type alkaloids, respectively^31^. Gelsechizines A (**69**) and B (**70**) with the usual three nitrogen atoms were isolated from the fruits of *G. elegans*. Differently, gelsechizine A was characterised with a methyl 1,2,6,7,12,12 b-hexahydroindolo[2,3-a]quinolizine-3-carboxylate core and an additional 4-methylpyridine unit located at C15. While, gelsechizine B featured a (*4S*,*4aS*,*13bS*,*14aS*)-methyl 3,4,4a,5,7,8,13,13b,14,14a-decahydro-4-methylindolo[2′,3′:3,4]pyrido [1,2-*b*][2,7]naphthyridine-1-carboxylate skeleton^34^ (Figure 6(A)).

### Sarpagine-type alkaloids

Sarpagine**-**type alkaloids feature an exocyclic (*E*)-ethylidene side chain and a cage-shaped scaffold that is made up of two bridged substructures, namely indole-fused azabicyclo[3.3.1]nonane and azabicyclo[2.2.2]octane. Of note, this type of alkaloids is especially high not only in *Gelsemium* genus, but also in *Gardneria*, *Rauwolfia,* and *Alstonia* genera (Apocynaceae)^35^^,^^36^. *epi*-Koumidine *N*_4_-oxide (**71**), isolated from the stems of *G. elegans*, had the presence of one more oxygen atom than that of *epi*-koumidine, indicating that the formation of a coordination bond was formed among oxygen and nitrogen atoms. It represented the first example of *N*_4_-oxide sarpagine-type alkaloid from the *Gelsemium* genus^18^ (Figure 6(B)).

### Gelsemine-type alkaloids

Gelsemine-type alkaloids with seven contiguous stereocenters bear a *spiro*-indoleone unit, a rare oxidised[3.2.1]bicyclic architecture, and a 1-methyl-3-vinylpyrrolidine ring compacted into a caged framework. (*4 R*)-19-Oxo-gelsevirine *N*_4_-oxide (7**2**), a *N*_4_-oxide derivative was isolated from the roots of *G. elegans*. Compared to typical gelsevirine, the vinyl group in gelsemine was replaced by an acetyl group, and the N1 atom was substituted by a methoxyl group in (*4 R*)-19-oxo-gelsevirine *N*_4_-oxide^20^ (Figure 6(C)).

### Other-type alkaloids

**Figure 7.**
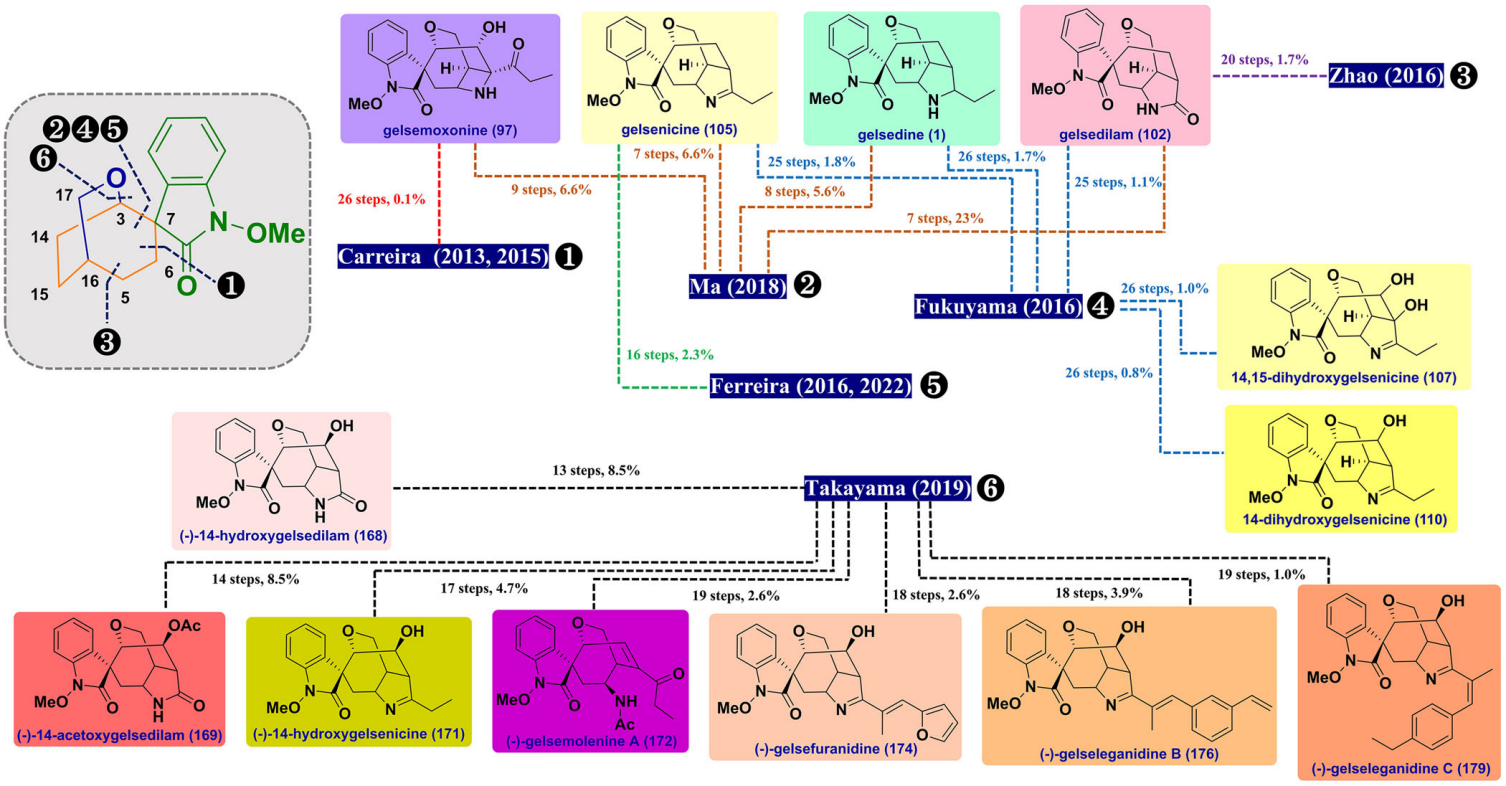
The chemical structures of novel other-type alkaloids.

10,11-Dimethoxy-*N*_1_-demethoxy-gelsemamide (**73**), 11-demethoxy-gelsemazonamide (**74**), geleganamide (**75**), and geleganidine B (**76**), four unusual MIAs, were isolated from the different parts of *G. elegans*. 10,11-Dimethoxy-*N*_1_-demethoxy-gelsemamide possessed a characteristic symmetrical dimeric N1-C2 *seco*-indole unit, whose skeleton might be derived from humantenine-type alkaloids. 11-Demethoxy-gelsemazonamide, geleganamide, and geleganidine B belonged to three rare dimeric derivatives of open-loop indole alkaloids. The difference between the three compounds was that the two formers were bridged by an azo group to form an aromatic azo-containing substructure, while the latter was connected by a biphenyl structure. The methoxy group at C11 in geleganidine B was missing in 11-demethoxy-gelsemazonamide that represented the first nonsymmetric aromatic azo-linked bisindole alkaloid^20^^,^^27^^,^^30^ (Figure 7).

## Pharmacological activity

In recent years, a large number of studies have proven that gelsemium MIAs exhibit extensive beneficial pharmacological activities, which primarily focus on analgesic, anti-tumour, anxiolytic, immunosuppressive, and anti-inflammatory aspects. Among general gelsemium MIAs, the activity evaluations of gelsemine and koumine are the most intensively studied. These pharmacological activities could be briefly summarised as follows (Table 2).

**Table 2.** The pharmacological effects of gelsemium MIAs in *vivo* and *in vitro* experiments.

Bioactivity	Compounds	Route	*In vivo* doses (mg/kg)	*In vitro* doses (μM)	Possible mechanisms	*In vivo* model (s)	*In vitro* mode (s)	Reference
Analgesic activity	Koumine (**2**)	*s.c.*	0.4-10	–	Inducing TSPO allostery	Formalin-induced mice	–	^36^
			1.0	–		Collagen-induced rats	–	
			0.28	–		CCI-induced rats	–	
			10.0	–	Inducing TSPO allostery; increasing neurosteroid levels	Formalin-induced mice	–	^37^
			7.0	–		CCI-induced rats	–	
			0.28-7.0	–	Enhancing 3α-HSOR mRNA expression and bioactivity	CCI-induced rats	–	^38^
			0.28-7.0	–	Inhibiting glial activation; suppressing proinflammatory cytokines	Plantar incision-induced rats	–	^39^
			0.28-7.0	25.0-200.0	–	CCI-induced rats	LPS-induced BV2 cells	^40^
			0.28-7.0	25.0-100.0	Inhibiting astrocyte activation and pro-inflammatory cytokines; promoting autophagy; reducing apoptosis	CCI-induced rats	LPS-induced primary astrocytes	^41^
			0.28-7.0	–	–	STZ-injected rats	–	^42^
			0.28-7.0	–	Inhibiting microglial M1 polarisation and proinflammatory mediators via blocking the Notch-RBP-Jκ pathway	STZ-injected rats	–	^43^
			0.28-7.0	–	Reducing the clearance of koumine	STZ-injected rats	–	^44^
	gelsemine (**3**)	*i.t.*	0.03-10.0 (μg)	0.1-100.0	Activating spinal α3 glycine receptors	Formalin-induced rats	–	^45^
						Bone cancer-induced rats	–	
						L_5_/L_6_ SNL-induced rats	–	
		*-*	–	0.1-100.0	Modulating glycine receptors	–	Glycine receptors-expressed HEK293 cells	^46^
		*i.t.*	10.0 (μg)	0.001-0.1	Stimulating 3α-HSOR expression	L_5_/L_6_ SNL-induced rats	Primary neuronal cells	^47^
		*i.p.*	1.0-4.0	–	–	PSNL-induced mice	–	^48^
	*N*-desmethoxyhumantenine *N*_4_-oxide (**48**)	*i.p.*	1.0	–	–	Acetic acid-induced mice	- -	^17^
	gelstriamine A (**50**)		0.04, 0.2	–				
Antitumor activity	sempervirine (**6**)	*i.p.*	4.0, 8.0	1.0-8.0	Mediating autophagy and apoptosis; blocking Akt/mTOR pathway	Xenograft U251 cells bearing nude mice	U251 cells	^49^
		*i.p.*	1.0	0.1-1.0	Inducing apoptosis; blocking the cell cycle in the G1 phase; upregulating p53 and downregulating cyclin D1, cyclin B1, and CDK2; inactivating the Wnt/β-catenin pathway	Xenograft HepG2 cells bearing nude mice	HepG2 cells	^50^
		–	–	0.8-5.0	Degradating RNA polymerase I; disrupting ribosomal content; blocking MDM2 via inhibition of E2F1/pRB pathway	–	Testicular germ cell tumours cells	^51^
	14*β*-hydroxygelsedethenine (**17**)	–	–	8.3-90.3 (IC_50_)	–	–	NCI-H1975, PC9, NCI-H460, NCI-H661, and H292 cell lines	^18^
	gelseleganin C (**21**)	–	–	<10.0 (IC_50_)	–	–	A-549, SPC-A cells, 1D356, OC3, Tca8113, SACC83, and MEC1 cells	^16^
	11-methoxy-14,15-dihydroxygelsedine (**37**)	–	–	10.9-12.1 (IC_50_)	–	–	Hep-2, LSC-1, TR-LCC-1 and FD-LSC-1 cells	^22^
	geleganidine C (**46**)	–	–	16.1 (IC_50_)	–	–	PC-12 cells	^29^
	geleganidine B (**76**)	–	–	38.4 (IC_50_)	–	–	MCF-7 cells	
	norhumantenine A (**53**) and *N*_4_-demethyl-21-dehydrokoumine (**58**)	–	–	4.6-9.3 (IC_50_)	–	–	HL-60, SMMC-7721, A-549, MCF-7, SW480 and BEAS-2B cells	^30^
	koumine (**2**)	*p.o.*	2.0	25.0-125.0	Inducing cell apoptosis and suppressing glycolysis via inhibiting Akt/mTOR/HK2 pathway and promoting HK2 disassociation to VDAC-1 via targeting PDK1	Xenograft HCT116 cells bearing nude mice	HCT116 and HT29 cells	^46^
Anxiolytic activity	koumine (**2**)	*i.g.*	0.25-4.0	–	–	Mice	–	^53^
		*s.c.*	0.167-1.5	–	–	Rats	–	
		*s.c.*	0.167-1.5	–	Binding to TSPO protein; suppressing ACTH and CORT levels; increasing progesterone and allopregnanolone levels	Predatory sound stress-induced rats	–	^54^
	gelsemine (**3**)	*s.c.*	0.0001, 1.0 (μM)	–	–	Rats	–	^55^
		*i.p.*	0.4-10.0	–	Activating NLRP3 inflammasome; upregulating CREB and BDNF expressions	CUMS-induced mice	–	^56^
Anti-inflammatory activity	koumine (**2**)	*i.p.*	0.8-7.2	50.0-200.0	Blocking ROS/NF-κB/NLRP3 pathway	MSU-induced mice	LPS/ATP/MSU-induced BMDM and THP cells	^57^
	gelsekoumidine B (**16**)	–	–	33.2 (IC_50_)	–	–	LPS-induced RAW 264.7 cells	^20^
	gelsecorydines A (**25**), C (**27**), D (**28**) and E (**29**)	–	–	4.2-16.2 (IC_50_)	–	–	LPS-induced RAW 264.7 cells	^24^
	gelserancines B (**31**), C (**32**) and **D** (**33**)	–	–	50.0	–	CuSO_4_-induced zebrafish	–	^25^
	geleganimine B (**36**)	–	–	10.2 (IC_50_)	–	–	LPS-induced BV2 cells	^26^
	gelsechizines A (**69**) and B (**70**)	*-*	12.5, 25.0	12.5, 25	Inhibiting the recruitment of neutrophils and macrophages; reducing the secretion of TNF-α and IL-6	LPS-induced zebrafish	LPS-induced RAW 264.7 cells	^33^
	(*4R*)-19-oxo-gelsevirine *N*_4_-oxide (**72**) and 10,11-dimethoxy-*N*_1_-demethoxy-gelsemamide (**73**)	–	–	6.2, 12.2 (IC_50_)	–	–	LPS-induced RAW 264.7 cells	^19^
Anti-rheumatoid arthritis and immuno suppressive activities	gelsevirine (**77**)	*i.p.*	5.0	6.25, 50.0	Promoting the K48-ubiquitination of STING	STING-deficient mice and medial meniscus-operated mice	IL-1β-stimulated mouse primary chondrocytes	^58^
	koumine (**2**)	*p.o.*	0.6-15.0	–	Inhibiting B lymphocytes-induced immune complexes and secondary inflammation	CFA-induced Rats; Collagen-induced rats	–	^59^
		*p.o.*	0.6-15.0	25.0-100.0	Reducing astrocyte activation and pro-inflammatory cytokines	Collagen-induced rats	LPS-induced primary astrocytes	^60^
		*p.o.*	0.4-10.0	10.0-70.0	–	TD Ag NP-CGG-immunized C57BL/6J mice	LPS-induced B cells	^61^
		*p.o.*	4.0, 8.0	–	Regulating RORγt/Foxp3 expressions; correcting Th17/Treg immune imbalance	Collagen-induced mice	–	^62^

CCI: chronic constriction injury; CFA: complete Freund’s adjuvant; CUMS: chronic unpredictable mild stress; HSOR: hydroxysteroid oxidoreductase; *i.p.*: intraperitoneal; *i.t.*: intrathecal; LPS: lipopolysaccharide; MSU: monosodium urate; PSNL: partial sciatic nerve ligation; *s.c.*: subcutaneous; SNL: spinal nerve ligation; STZ: streptozocin; TSPO: translocator protein 18 kDa.

### Analgesic activity

Several research has indicated that gelsemium MIAs exert analgesic properties *in vivo* and *in vitro*. Koumine (**2**) is the most abundant MIA of *G. elegans*. Its treatment displayed efficient analgesic activity against inflammatory and neuropathic pain in a variety of rodent models^37^. Mechanistic studies revealed that koumine could function as a high-affinity ligand that interacted with translocator protein 18kda positive (TSPO) protein in microglia, thereby inducing TSPO allostery^37^^,^^38^. TSPO allostery triggered the biosynthesis of neurosteroids, such as allopregnanolone in the spinal cords, which mediated the reduction of neuropathic pain^39^. Moreover, *in vivo* and *in vitro* studies also found that koumine enabled to inhibit the production of proinflammatory cytokines and glial activation^40^^,^^41^. Because TSPO is the typical marker of activated microglia, we conjectured that this anti-inflammatory action of koumine might also be related to TSPO allostery. Besides, koumine also increased astrocyte autophagy occurrence and decreased astrocyte-related inflammation, which was the mechanistic basis for its analgesic activity^42^. In terms of improvement in diabetic neuropathic pain (DNP), the neuropathic pain behaviour and the injury of axon and myelin sheath of the sciatic nerve were greatly ameliorated in streptozocin (STZ)-induced diabetes rats after subcutaneous treatment with koumine (0.28, 1.4, and 7.0 mg/kg, for 7 days)^43^. Its analgesic effects could be attributed to the modulation of spinal microglial M1 polarisation and proinflammatory mediators via inhibiting the Notch-RBP-Jκ signalling pathway^44^. Moreover, the study on pharmacokinetics indicated that koumine elimination was decreased in STZ-induced rats, suggesting koumine was retained for the treatment of DNP *in vivo*^45^. Gelsemine (**3**), is the principal active alkaloid from *G. sempervirens*. Like koumine, gelsemine also was able to inhibit nociceptive pain and tonic pain in different pain models. Its mechanism for analgesic activity was that gelsemine might serve as a potential α3 glycine receptor (α3-GlyR) agonist to modulate the function of spinal α3-GlyRs and stimulate the biosynthesis of allopregnanolone through upregulation of the mRNA expression of 3α-hydroxysteroid oxidoreductase^46–48^. Where after, gelsemine’s analgesic effect was reported in partial sciatic nerve-ligated mice as its administrations (2.0 and 4.0 mg/kg, *i.p.*) alleviated both neuropathic pain and sleep disturbance, and upregulated c-Fos expression in the neurons of the anterior cingulate cortex^49^. Additionally, intraperitoneal *N-*desmethoxyhumantenine *N*_4_-oxide (**48**) treatment at lower doses of 0.04 and 0.2 mg/kg alleviated acetic acid intraperitoneal injection-induced writhing of mice with inhibition rates of 67.6 and 76.1%, respectively, which were even stronger than those of the positive control, morphine. Additionally, gelstriamine A (**50**) treatment (1.0 mg/kg, *i.p.*) showed potent analgesic activity with a reduced rate of 64.7% in the same model^18^.

### Antitumor activity

Sempervirine (**6**) displayed significant anti-cancer effects in several *in vitro* and *in vivo* cancer models. In glioma U251 cells, sempervirine (1.0, 4.0, and 8.0 μM) could inhibit tumour cell proliferation, suppress colony formation, and cause cellular G2/M phase arrest. Sempervirine also could promote the occurrence of cellular autophagy via the blockade of the Akt/mTOR signalling pathway. An *in vivo* experimental result showed that sempervirine (4.0 and 8.0 mg/kg, *i.p.*, for 28 days) significantly decreased the growth of glioma cancer by 44.76% and 61.26%, respectively^50^. In human hepatocellular carcinoma, sempervirine (0.1, 0.5, and 1.0 μM) induced HepG2 cells apoptosis and blocked the cell cycle in the G1 phase, accompanied by the upregulation of p53 and the downregulation of cyclin D1, cyclin B1, and CDK2. In the xenograft nude mice model, sempervirine treatment (1 mg/kg, *i.p.*, for 18 days) substantially inhibited tumour growth and enhanced the anti-tumour effect of sorafenib. The underlying mechanism was involved in the inactivation of the Wnt/β-catenin pathway^51^. Sempervirine treatment (0.8–5.0 μM) triggered cell death in both *p53*-wildtype and *p53*-null testicular germ cell tumours (TGCT) cells. Mechanistically, sempervirine could translocate into the nucleus, where it bound rRNA to induce RNA polymerase I (RNA Pol I) degradation and disrupt ribosomal content. This cascade further mediated the MDM2 block to kill cancer cells via concomitant inhibition of the E2F1/pRB pathway^52^. 14β-Hydroxygelsedethenine (**17**) exhibited cytotoxicity against NCI-H1975, PC9, NCI-H460, NCI-H661, and H292 cell lines, with IC_50_ values ranging 8.3–90.3 μM, respectively^19^. Gelseleganin C (**21**) displayed significant cytotoxic activities against A549, SPC-A, 1D356, OC3 Tca8113, SACC83, and MEC1 cell lines, with IC_50_ values less than 10 μM^17^. 11-Methoxy-14,15-dihydroxygelsedine (**37**) displayed moderate cytotoxic activity with IC_50_ values of 12.1, 11.7, 10.9, and 11.4 μM towards Hep-2, LSC-1, TR-LCC-1, and FD-LSC-1 cell lines, respectively^23^. Geleganidine C (**46**) displayed a growth inhibitory effect against PC-12 cells with an IC_50_ value of 16.1 μM, while geleganidine B (**76**) showed weak activity against MCF-7 cells, with an IC_50_ value of 38.4 μM^30^. Norhumantenine A (**53**) and *N*_4_-demethyl-21-dehydrokoumine (**58**) exhibited moderate cytotoxicity against the six human tumours HL-60, SMMC-7721, A-549, MCF-7, SW480, and BEAS-2B cell lines, with IC_50_ values in the range 4.6–9.3 μM^31^. Our *in vivo* and *in vitro* studies of colorectal cancer have reported that koumine (**2**) displayed an antitumor effect. Koumine could induce apoptosis and suppress glycolysis via inhibiting Akt/mTOR/HK2 pathway and promoting the disassociation of HK2 to VDAC-1 via interaction with PDK1^53^.

### Anxiolytic activity

Several pieces of studies have shown that gelsemium MIAs play an effective role in anxiolytic activity. Koumine (**2**) administration by gavage (0.25, 1, and 4 mg/kg) exhibited anxiolytic-like properties in the open-field tests of ICR mice. Additionally, subcutaneous koumine treatment at the same concentration released anti-punishment action like diazepam in the Vogel conflict test of rats^54^. Koumine treatment (0.167, 0.5, and 1.5 mg/kg, *s.c.*) mitigated anxiety-like behaviour of acute predatory sound stress-induced rats in the open field test and elevated plus maze test. Its treatment also led to an increase in progesterone and allopregnanolone levels in the prefrontal cortex and hippocampus and a decrease in ACTH and CORT levels in plasma, suggesting that its anxiolytic mechanism was related to mediating neurosteroids-HPA axis^55^. In 2013, an *in vitro* study performed in rats reported that gelsemine (**3**) treatment at low doses (0.0001 and 1 μM, *i.p.*, for 7 days) significantly improved anxiety-specific parameters, some of which even approached the activity of the positive control, benzodiazepine diazepam^56^. Also, gelsemine when administered to chronic unpredictable mild stress-induced ICR mice by gavage (0.4, 2.0, and 10.0 mg/kg, for 9 days) substantially altered anxiety-like behavioural performance via inhibiting NLRP3-inflammasome and upregulating CREB and BDNF expression in the hypothalamus^57^.

### Anti-inflammatory activity

Koumine (**2**) treatment (0.8, 2.4, and 7.2 mg/kg) dramatically decreased the production of IL-1β in the MSU-induced peritonitis mice model, consistent with an inhibitory effect on NLRP3 inflammasome activation and NF-κB pathway. Additionally, an *in vivo* study confirmed that koumine treatment (50, 100, and 200 μM) antagonised inflammation in LPS-primed macrophages treated with ATP or MSU via blockage of the NF-κB/NLRP3 signalling pathway^58^. Gelsekoumidine B (**16**) exhibited concentration-dependent inhibition of LPS-induced NO production in RAW 264.7 macrophage cells, with an IC_50_ value of 33.2 μM (indomethacin was used as a positive control, IC_50_=23.1 μM)^21^. Gelsecorydines A (**25**), C (**27**), D (**28**), and E (**29**) exhibited a dose-dependent inhibition on LPS-caused NO production in macrophage RAW 264.7 cells, with IC_50_ values of 14.7, 16.2, 13.7 and 4.2 μM, respectively, some of which were approximately 1.5- to 15.8-fold stronger than the positive control, indomethacin, with an IC_50_ value of 21.0 μM^25^. Gelserancines B (**31**), C (**32**), and D (**33**) were evaluated for their anti-inflammatory effects *in vivo*, using dexamethasone as the positive control. Gelserancines B-D (50 μM) significantly reduced the neutrophil number in inflammatory sites in zebrafish acute inflammatory models which were induced by tail fin injury or CuSO_4_^26^. Geleganimine B (**36**) decreased LPS-induced NO production in BV2 cells, with an IC_50_ value of 10.2 μM, suggesting the reduction of the pro-inflammatory state^27^. Gelsechizines A (**69**) and B (**70**) (12.5 and 25.0 μM) exerted potent anti-inflammatory effects on LPS-induced zebrafish by inhibiting the recruitment of neutrophils and macrophages. Furthermore, the two compounds were shown to significantly inhibit the secretion levels of TNF-α and IL-6 in LPS-stimulated RAW 264.7 macrophage cells. SAR study showed that the existence of *β*-N-acrylate moiety might be an important factor in their anti-inflammatory effect.^34^. (*4 R*)-19-Oxo-gelsevirine *N*_4_-oxide (**72**) and 10,11-dimethoxy-*N*_1_-demethoxy-gelsemamide (**73**) exhibited a dose-dependent inhibitory effect on LPS-induced NO production in RAW 264.7 macrophage cells, with IC_50_ values of 6.2 and 12.2 μM, respectively (indomethacin was used as the positive control, IC_50_=21.7 μM)^20^.

### Anti-rheumatoid arthritis and immunosuppressive activities

Gelsevirine (**77**) has been well described to have an excellent anti-osteoarthritis effect. In IL-1β-stimulated mouse primary chondrocytes, its treatment (6.25 and 50.0 μM) dose-dependently enhanced cell viability and mitigated cell apoptosis. Moreover, it could downregulate the mRNA expression of MMPs and inflammatory factors and upregulate the mRNA expression of Col2A and IL-10 via suppression of STING activation. In an *in vivo* experiment, chronic exposure to gelsevirine (5.0 mg/kg, *i.p.*, every 3 days for 10 weeks) could markedly reduce OARSI scores and MMP13 expression levels and increased cartilage area and Col2A expression levels in STING-deficient mice and the destabilisation of the medial meniscus-operated mice. Its latent mechanism was in conformity with the promotion of the K48-ubiquitination of STING^59^. Recent studies on collagen-induced rats of arthritis disclosed that treatment with koumine (**2**) alone (0.6, 3.0, or 15.0 mg/kg, *i.g.*, for 10 days) exerted an inhibitory effect on joint pain, that concomitantly occurred with an improvement in the arthritis index scores, mechanical allodynia and volume of injected hind paw as well as the destruction of bone and cartilage. Moreover, koumine effectively ameliorated the production of proinflammatory cytokines in joint tissues and astrocyte activation in the spinal cords. Studies on its antirheumatic mechanism revealed that koumine suppressed the secretion of anti-CII antibody, which was produced by B lymphocytes and could damage joints via the occurrence of the inflammatory response^60^^,^^61^. In 2022, koumine treatment decreased T cell-dependent and T cell-independent B cell immune response *in vivo* and *in vitro*, which might be an alternative mechanism for its anti-rheumatoid arthritis bioactivity^62^. Such evidence was also demonstrated by an *in vivo* study in which koumine pre-treatment (4.0 and 8.0 mg/kg, *p.o.*, for 3 weeks) exhibited a therapeutic effect on CIA in mice through regulation of RORγt/Foxp3 signal pathway and modulation of Th17/Treg immune imbalance^63^.

## Total synthetic chemistry

**Figure 8.**
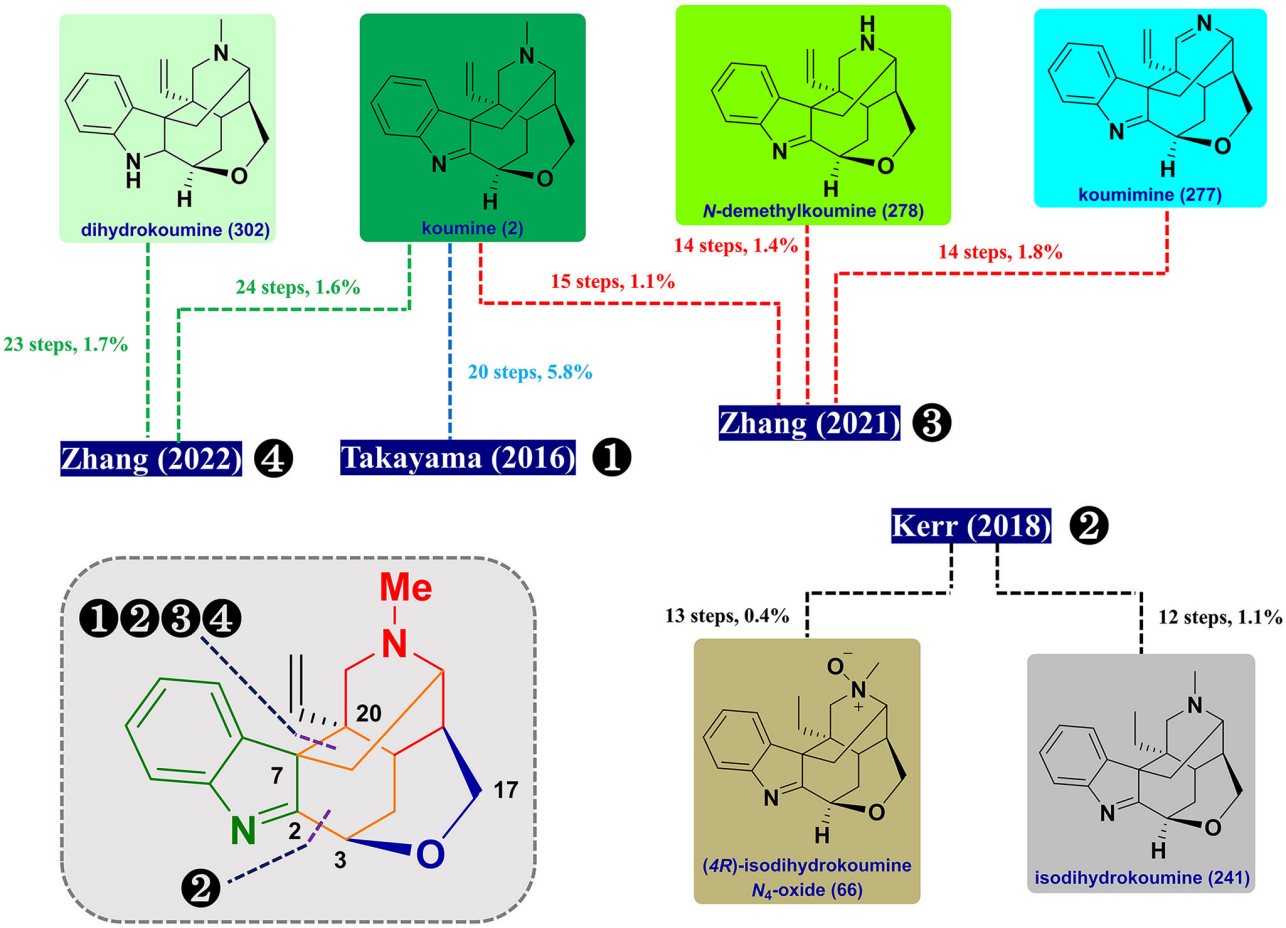
Schematic summary of previous total syntheses of gelsedine-type alkaloids (2013–2022).

**Figure 9.**
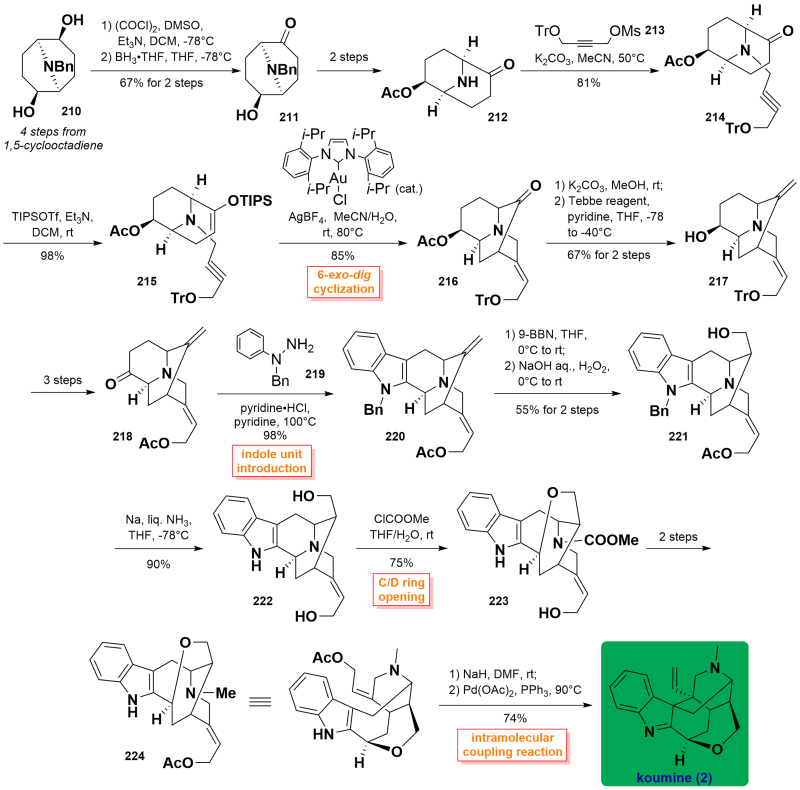
Schematic summary of previous total syntheses of koumine-type alkaloids (2013–2022).

Due to profuse and diverse effects along with their distinctive chemical structures, tremendous efforts have been devoted to synthetic approaches towards the total synthesis of gelsemium MIAs. Gelsemine and koumine-type alkaloids, the flagship members of gelsemium, have been widely studied by several synthetic chemists. Conversely, yohimbane-type alkaloids with relatively simple structures have received relatively less attention. According to the different molecular skeletons of gelsemium MIAs, these synthetic approaches were classified as follows. (Figure 8 and 9)

### Total syntheses of gelsedine-type alkaloids

#### Carreira’s total synthesis of (±)-gelsemoxonine (2013, 2015)

**Scheme 1:**
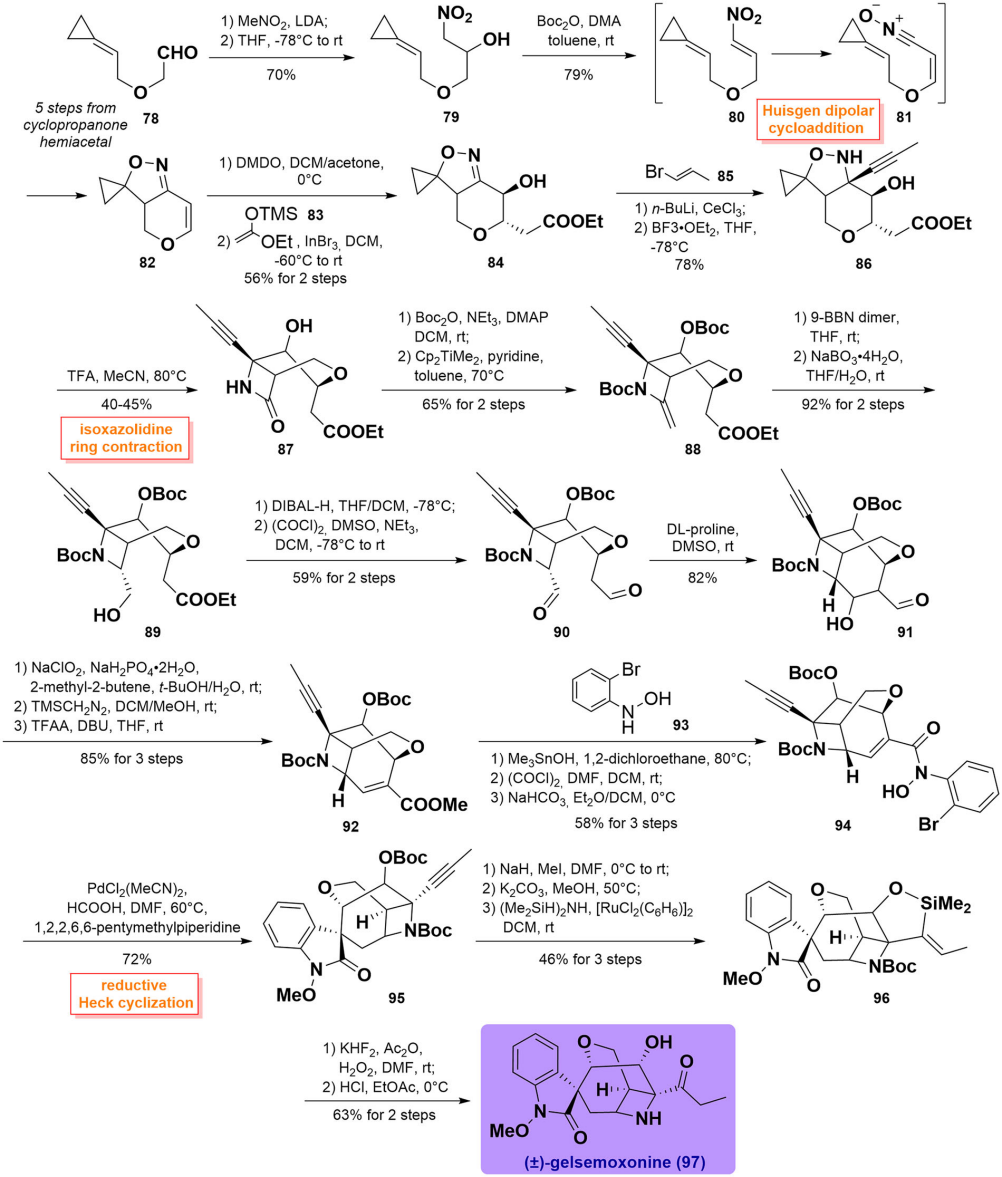
Carreira’s total synthesis of (±)-gelsemoxonine.

Carreira and co-workers achieved the total synthesis of (±)-gelsemoxonine using a ring contraction approach of a spirocyclopropane isoxazolidine to introduce the β-lactam intermediate, providing access to the unusual azetidine^64^^,^^65^. They began with aldehyde **78** (5 steps from cyclopropanone hemiacetal), which was converted into nitro-alcohol **79** in high yield through Henry reaction. Then, it was prepared to isoxazoline **82** via elimination followed by intramolecular Huisgen dipolar cycloaddition induced by Boc_2_O/DMAP between nitrone and alkene. It was then treated with DMDO to get an epoxy intermediate, which was attacked by ketene silyl acetal **83** via nucleophilic addition to generate alcohol **84**. Its reaction with 1-bromo-1-propene **85** installed a 1-propynyl moiety furnished diastereomeric oxazolidine **86** using anhydrous CeCl_3_ and BF_3_·OEt_2_. **86** underwent the key ring contraction rearrangement to build the isoxazolidine ring in **87** employing TFA in 40–45% yield. After Boc group protection, treatment of the carbonyl group in **87** with Petasis’ olefination generated olefin **88** *in situ*, followed by a concomitant hydroboration reaction to yield the desired primary alcohol **89** with high diastereoselectivity. Subsequently, **89** was transformed to dialdehyde **90** via reduction with DIBAL-H along with oxidation of the diol under Swern conditions. Furthermore, the 7-membered ring was set up through an intramolecular aldol reaction catalysed by DL-proline, thus yielding aldol **91** as a single diastereomer. **91** was further advanced to unsaturated ester **92** via a multistep reaction sequence, including Pinnick oxidation, esterification, and hydroxyl elimination. Through hydrolysis using Me_3_SnOH, the condensation of **92** with *N*-(2-bromophenyl)hydroxylamine **93** proceeded to forge aryl bromide **94**, whose exposure to reductive Heck conditions afforded oxindole **95** as a single diastereoisomer in 72% yield. After esterification and selective removal of the Boc group using K_2_CO_3_, the corresponding alcohol was immediately subjected to hydrosilylation employing [RuCl_2_(C_6_H_6_)]_2_ as catalysis to deliver vinylsilane **96** in 46% yield over 3 steps. Finally, Tamao-Fleming oxidation followed by removal of the *N*-Boc group using HCl provided the natural product (±)-gelsemoxonine (**97**). (Scheme 1)

#### Fukuyama’s total syntheses of (-)-gelsenicine, (-)-gelsedine, (-)-gelsedilam, (-)-14-hydroxygelsenicine, and (-)-14,15-dihydroxygelsenicine (2016)

**Scheme 2:**
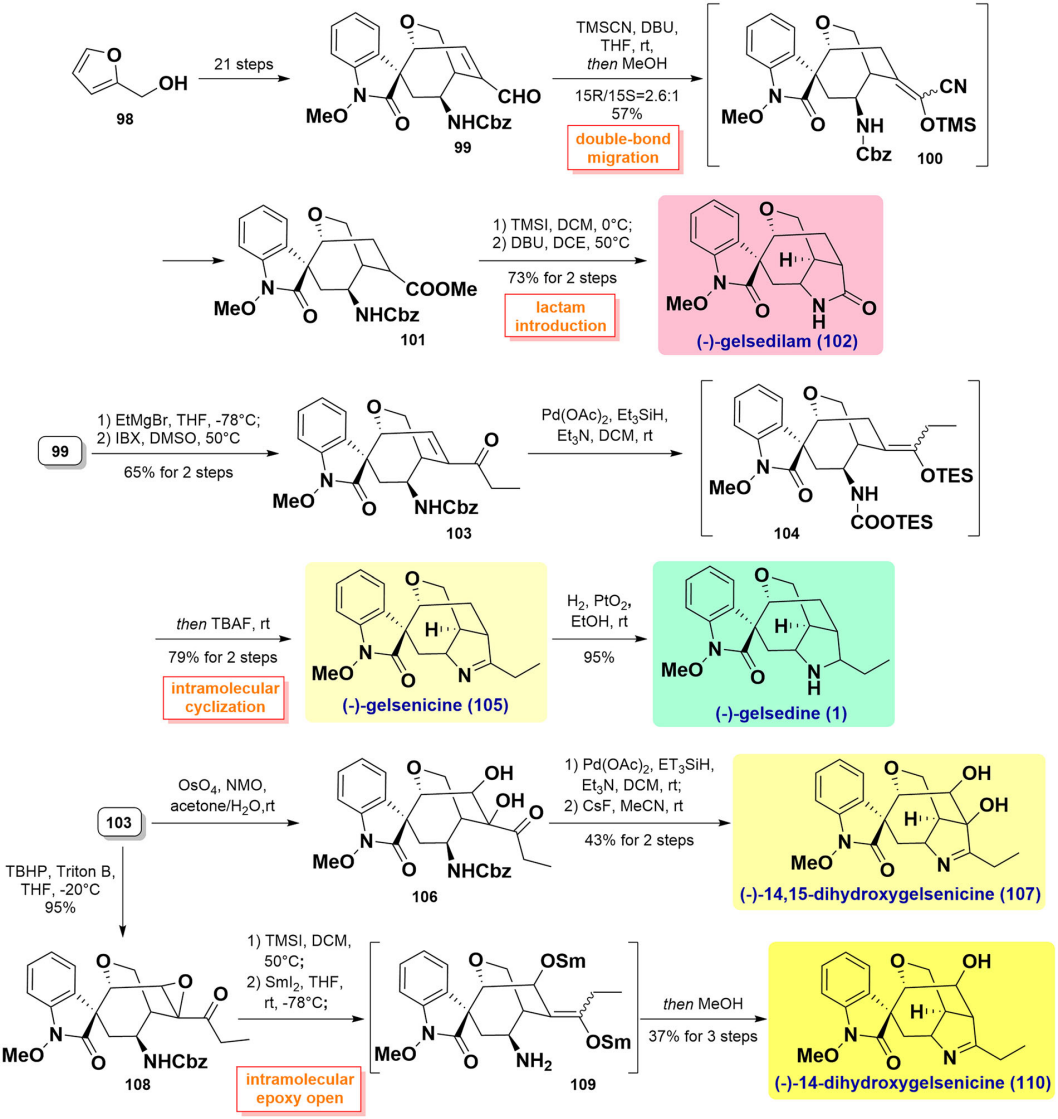
Fukuyama’s total syntheses of five gelsedine-type alkaloids.

In Fukuyama’s study, a flexible and unified synthetic route was developed to construct a library of gelsedine-type alkaloids through an enal intermediate bearing a versatile core structure^66^. The key intermediate aldehyde **99** was synthesised from furfuryl alcohol **98** in 21 steps as reported in earlier Fukuyama’s study^67^. TMSCN/DBU-mediated redox isomerisation reaction led to the formation of acyl cyanide derivative **100**. It was followed by a nucleophilic reaction with MeOH to afford methyl ester **101** (15 *R*/15*S* = 2.6:1), and the desired 15 *R* isomer was isolated in 57% yield. Deprotection of the Cbz group by TMSI and subsequent *N*-acylation reaction in the presence of DBU in DCE completed the intermolecular cyclisation, thereby furnishing (-)-gelsedilam (**102**). In parallel, the instalment of the ethyl group onto **99** using EtMgBr followed by IBX-induced hydroxyl oxidation afforded another key intermediate carbonyl **103**. Then, Pd(OAc)_2_-catalysed conjugate reduction of the unsaturated ketone with *in situ* trapping as its silyl enol ether at base condition yielded the resulting **104**. It was exposed to TBAF, and then the liberated amine and ketone were facilely cyclized to achieve the total synthesis of (-)-gelsenicine (**105**). It was further subjected to hydrogenation reaction by Adams’ catalyst to afford (-)-gelsedine (**1**). In addition, the exposed double bond of **103** was oxidised by catalytic OsO_4_/NMO in acetone/H_2_O to yield diol **106**, which underwent similar two-step Pd(OAc)_2_*/*Et_3_SiH treatment and concomitant dehydrative cyclisation yielded (-)-14,15-dihydroxygelsenicine (**107**) in 43% yield over 2 steps. Meanwhile, the epoxy moiety was diastereoselectively introduced in treating TBHP/Triton B on C14 and C15 in **103** to afford epoxy **108**. After the loss of the Cbz group by TMSI in DCM, the treatment of this substrate with reductive SmI_2_ in THF at −78 °C allowed the reduction of α,β-epoxy ketone to β-hydroxy ketone, further forming the samarium enolate **109**. When it was protonated in MeOH, the Schiff’s base was simultaneously synthesised, eventually affording (-)-14-hydroxygelsenicine (**110**) in 37% yield over 3 steps. (Scheme 2)

#### Zhao’s total synthesis of gelsedilam (2016)

**Scheme 3:**
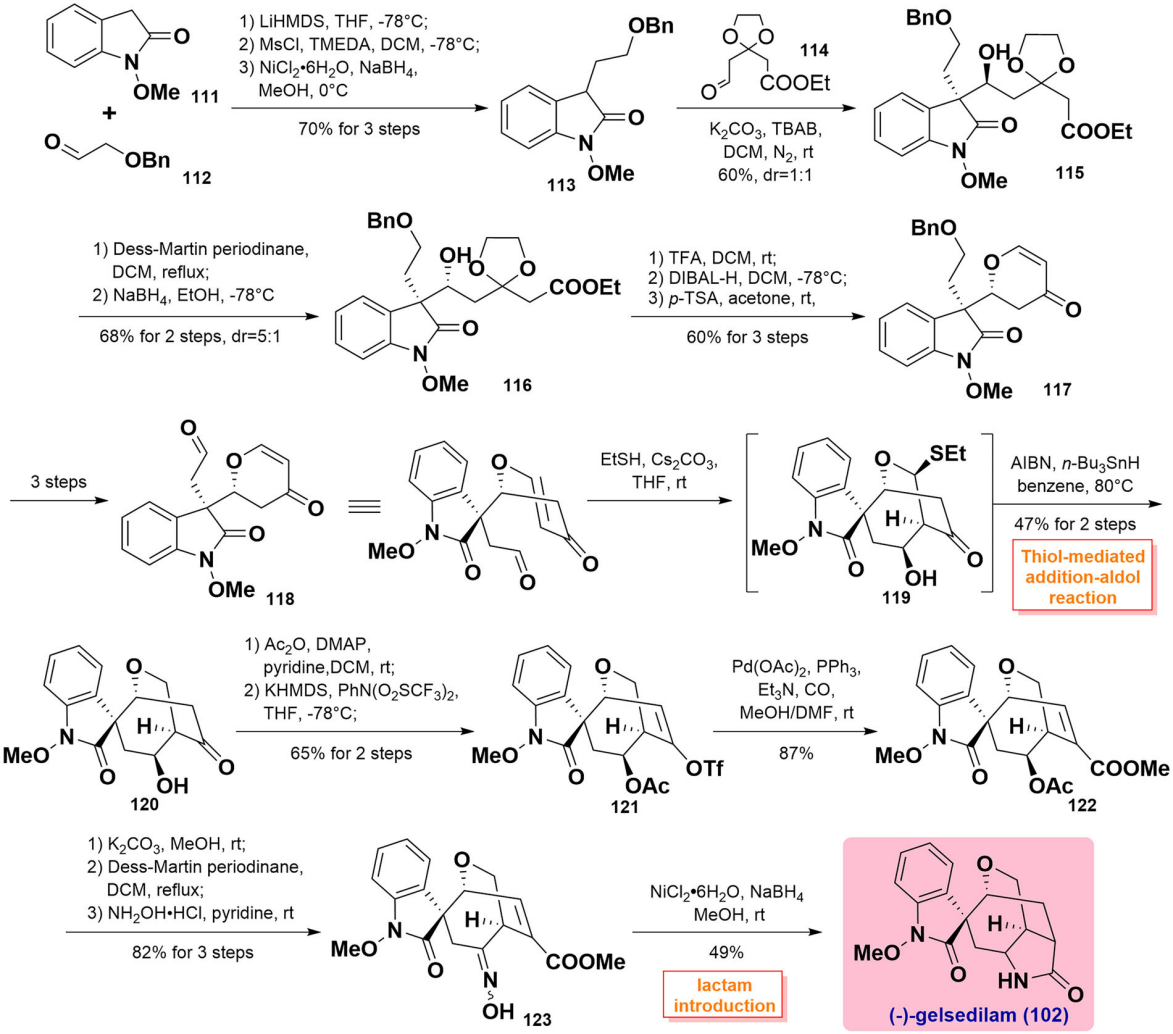
Zhao’s total synthesis of gelsedilam.

In 2016, Zhao and co-workers described the total synthesis of gelsedilam, by utilising a highly thiol-mediated diastereoselective conjugate addition-aldol reaction to construct the oxabicyclo[3.2.2]nonane ring system^68^. In the beginning, C3-substituted oxindole **113** was synthesised from *N*-OMe oxindole **111** and 2-(benzyloxy)acetaldehyde **112** via a sequence of aldol condensation, acylation, and reduction reaction in 70% yield over 3 steps. The following aldol reaction between **113** and aldehyde **114** occurred to construct compound **115** (dr = 1:1) using a mild base of K_2_CO_3_. Oxidation using Dess-Martin periodinane (DMP) and re-reduction subsequent were performed to switch the β-hydroxyl group to the α-hydroxyl group, and the diastereoselective ratio was noticeably improved from 1:1 to 5:1. Then, an intramolecular condensation employing TFA occurred to smoothly deliver lactone **116**, which was further selectively reduced by DIBAL-H at −78 °C and removed the ketal group by *p*-TSA in acetone to yield pyrone **117** in 60% yield over 3 steps. Next, aldehyde **118** was synthesised over 3 steps. Cs_2_CO_3_-promoted Michael addition of thiol and conjugate addition-aldol reaction as key steps produced thiolated **119**, which was removed from the thiolate group in the presence of AIBN/*n*-Bu_3_SnH in benzene yielded the corresponding **120** as a single diastereoisomer in moderate yield. Upon acetylation protection, the carbonyl group in **120** was transformed into triflyl enol in **121** by treatment with KHMDS. Subsequent Pd(OAc)_2_-catalysed carbonylation reaction in the presence of CO obtained unsaturated ester **122**. Deprotection of the acetate group along with DMP oxidation furnished the ketone product, which was treated by hydroxylamine hydrochloride to obtain oxime **123**. It was further elaborated to complete the reduction with NaBH_4_ with the use of NiCl_2_ and *in situ* lactam cyclisation in one pot process to yield the final gelsedilam (**102**). (Scheme 3)

#### Ferreira’s total synthesis of (-)-gelsenicine (2016, 2022)

**Scheme 4:**
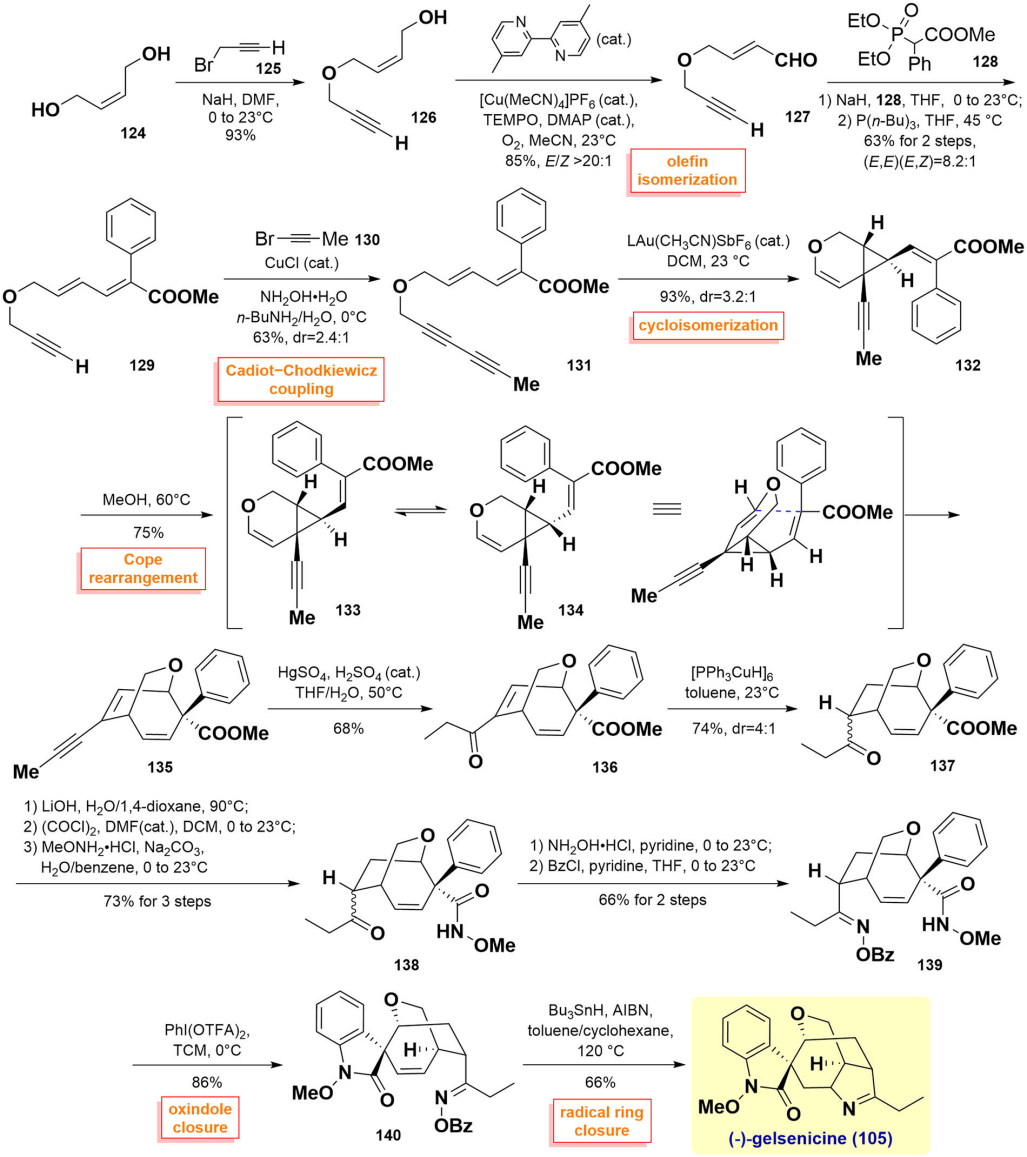
Ferreira’s total synthesis of (-)-gelsenicine.

Ferreira and co-workers reported the shortest approach towards the total synthesis of gelsenicine in 13 steps^69^^,^^70^. Their synthesis commenced with the alkylation of (*Z*)-but-2-ene-1,4-diol **124** with 3-bromoprop-1-yne **125,** along with Cu-catalysed oxidation and olefin isomerisation to forge aldehyde **127** (*E*/*Z* > 20:1) in satisfactory yield over 3 steps. Then, it underwent a Horner-Wadsworth-Emmons olefination with phosphonate **128** and phosphine-mediated alkene *E*/*Z* isomerisation to synthesise (*E*,*E*)-dienyne **129** in a high (*E*,*E*)/(*E*,*Z*) ratio of 8.2:1. Subsequent Cadiot-Chodkiewicz coupling of **129** with 1-bromo-1-propyne **130** allowed access to (*E*,*E*)-diyne **131**. Under the optimised condition, Au-catalysed cycloisomerization provided the resulting product **132** with an outstanding yield in a 3.2:1 dr ratio, whereupon a strain-release Cope rearrangement was performed to obtain bicycle **135** in MeOH at 60 °C in 75% yield. Regioselective Kucherov alkyne hydration using HgSO_4_/H_2_SO_4_ catalysis then transformed **135** into enone **136**. It was directly subjected to conjugate reduction to afford ketone **137** (dr = 4:1) using Stryker’s reagent, followed by a series of hydrolysis, acyl chloride, and amidation, thereby generating amide **138** with an overall yield of 73%. Oxime formation with hydroxylamine and benzoylation proceeded to install the oxindole unit, giving benzoyl-oxime **139**. The oxindole moiety was then assembled through a ring closure of amide using PhI(OTFA)_2_ in TCM at 0 °C, thus giving rise to oxindole **140**. Finally, the radical ring closure between benzoyl-oxime and olefin using Bu_3_SnH/AIBN at 120 °C successfully favoured (-)-gelsenicine (**105**). (Scheme 4)

#### Ma’s total syntheses of (-)-gelsedilam, (-)-gelsenicine, (-)-gelsedine, and (-)-gelsemoxonine (2018)

**Scheme 5:**
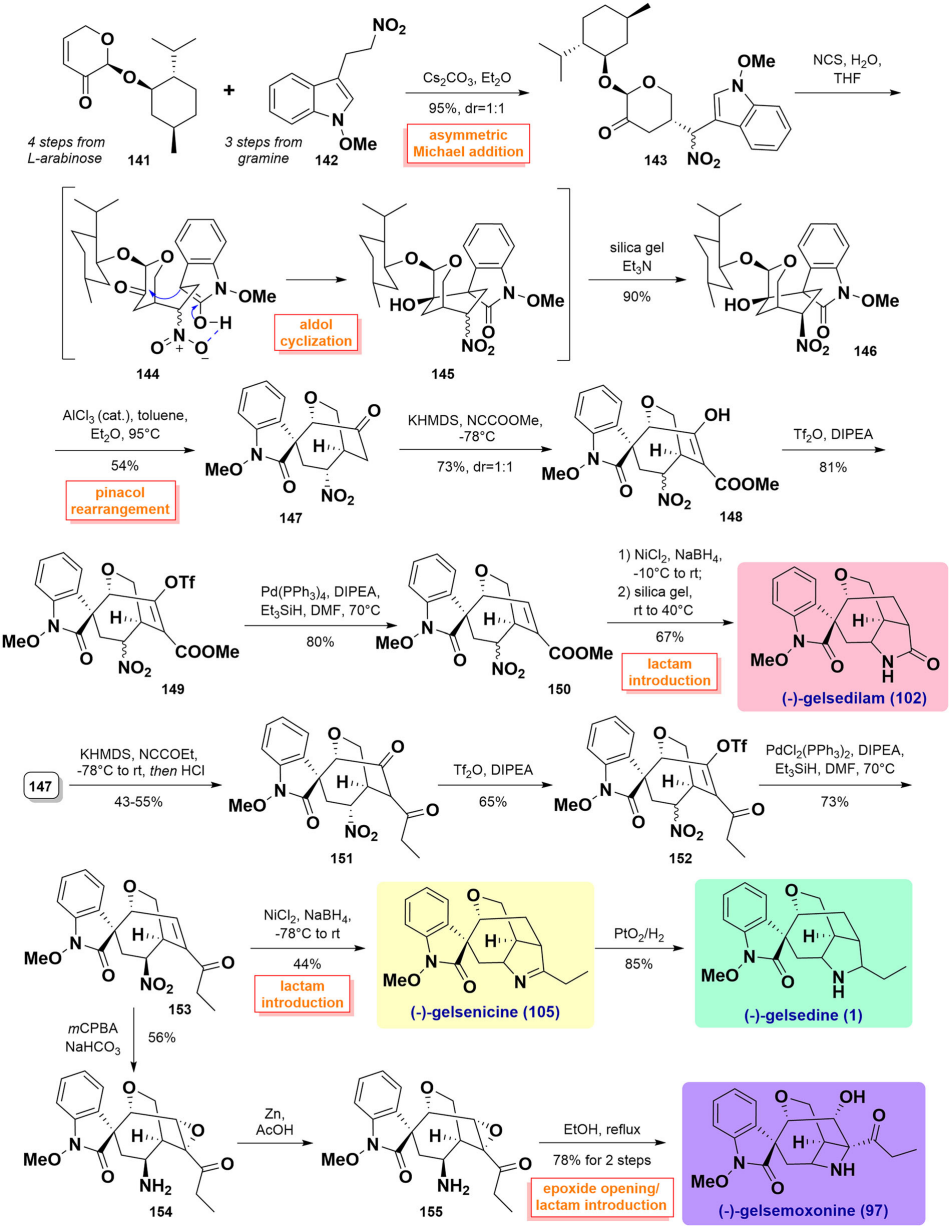
Ma’s total syntheses of four gelsedine-type alkaloids.

In 2018, Ma and co-workers developed and implemented a short total synthesis approach for four gelsedine-type alkaloids^71^. The synthesis started with an asymmetric Michael addition reaction of dihyropyranone **141** (4 steps from *L*-arabinose) and indole **142** (accessible from gramine in 3 steps), affording a 1:1 diastereoisomeric mixture of indolone **143**. After the formation of indolone upon treatment with NCS/H_2_O, subsequent aldol cyclisation smoothly established the oxabicyclo[3.2.2]nonane skeleton in **146** as a single isomer in 90% yield. Bicyclic **146** underwent an oxonium ion-induced pinacol rearrangement in toluene/Et_2_O and heating under AlCl_3_ catalysis to afford the key intermediate **147**. Then, the reaction of **147** with Mander’s reagent allowed the introduction of methyl formate at the α-carbon of the carbonyl group. The concomitant isomerisation of the α-position of the nitro group led to the formation of enol **148** as a 1:1 diastereomeric mixture. Subsequently, it was transformed into the corresponding enol triflate **149** under the conditions of Tf_2_O/DIPEA. Reductive removal of the triflate group using Pd(PPh_3_)_4_/Et_3_SiH afforded the corresponding α,β-unsaturated methyl ester **150**. Next, (-)-gelsedilam (**102**) was synthesised following Zhao’s approach as illustrated in Scheme 3. In parallel, treatment **147** with KHMDS and freshly distilled NCCOOEt provided diketone **151** as a single diastereomer in 4 3 ∼ 55% yield after quenching with HCl. **151** underwent the same synthetic procedure as **150** to afford α,β-unsaturated methyl ester **153**. Similarly, NiCl_2_/NaBH_4_-mediated nitro reduction accompanied by an intramolecular Schiff base formation provided (-)-gelsenicine (**105**). Furthermore, (-)-gelsedine (**1**) was synthesised via catalytic hydrogenation of **105** with PtO_2_ in the hydrogen atmosphere. In addition, the stereoselective epoxidation of **153** with *m*CPBA was followed by reduction of the nitro group with Zn in AcOH and furnished amino **155**. Epoxide opening reaction in boiling ethanol and intramolecular cyclisation yielded the desired (-)-gelsemoxonine (**97**). (Scheme 5)

#### Takayama’s unified total syntheses of (-)-14-hydroxygelsenicine, (-)-14-hydroxygelsedilam, (-)-14-acetoxygelsedilam, (-)-gelsemolenine A, (-)-gelsefuranidine, (-)-gelselegandine and (-)-gelselegandine C (2019)

In 2019, Takayama and co-workers achieved the first concise and collective asymmetric total synthesis of (-)-gelsemolenine A and five gelsedine-type alkaloids with a hydroxy group at C14^72^. The synthesis began with the stereoselective alkylation reaction of *γ*-lactone (*S*)-**156** with alkyl iodide **157** using LiHMDS to obtain compound **158**. It was subjected to ring-closing metathesis using Hoveyda-Grubbs II catalyst to forge cycloheptene **160**. Aminolysis of the *γ*-lactone in **159** under the influence of PMBNH_2_/DIBAL led to amide **160** in 99% yield. It was transformed into the corresponding enone **161** over 3 steps, which underwent an intramolecular aza-Michael addition reaction to give bicyclic **162** under LiHMDS condition. Selective 1,4-reduction of the enone in **162** employing L-Selectride and successive exposure to McMurry reagent generated the key enoltriflate **163** in quantitative yield. Pd(OAc)_2_-catalysed carbonylation cross-coupling between **163** and *N*-(2-bromophenyl)-*O*-methylhydroxylamine **164** at CO atmosphere constructed amide **165**. After cleavage of the TBDPS group, a Heck coupling reaction was performed to form *spiro*-*N*-methoxyoxindole **166** with high stereoselectivity, which upon an intramolecular alkoxymercuration-demercuration reaction gave alcohol **167**. Removal of the *p*-methoxybenzyl group by TFA in anisole achieved the total synthesis of (-)-14-hydroxygelsedilam (**168**) in 77% yield. It was acetylated to yield (-)-14-acetoxygelsedilam (**169**), whose lactam was protected by the Boc group and then treated with ethyl magnesium bromide to install an ethyl moiety, thus yielding the resulting **170**. It was deprotected using TFA to prepare (-)-14-hydroxygelsenicine (**171**) in 56% yield over 3 steps. (-)-Gelsemolenine A (**172**) was accessed by acetylation of **171** followed by treatment with aqueous HCl in methanol. Concurrently, **171** was condensed with 2-furaldehyde **173** and 3-vinylbenzaldehyde **176** under acidic conditions in 1,2-dichloroethane to obtain (-)-gelsefuranidine (**174**) and (-)-gelselegandine (**176**), respectively. Following a similar procedure, the condensation of **171** with 4-ethylbenzaldehyde **177** easily gave the product **178** having an *E*-configuration C19-C1′ double bond. Finally, photocatalytic riboflavin altered the olefin of *E*/*Z* configuration, and then led to the first total synthesis of (-)-gelselegandine C (**179**), albeit in 23% yield. (Scheme 6)

Scheme 6Takayama’s unified total syntheses of six gelsedine-type alkaloids.

**Figure:**
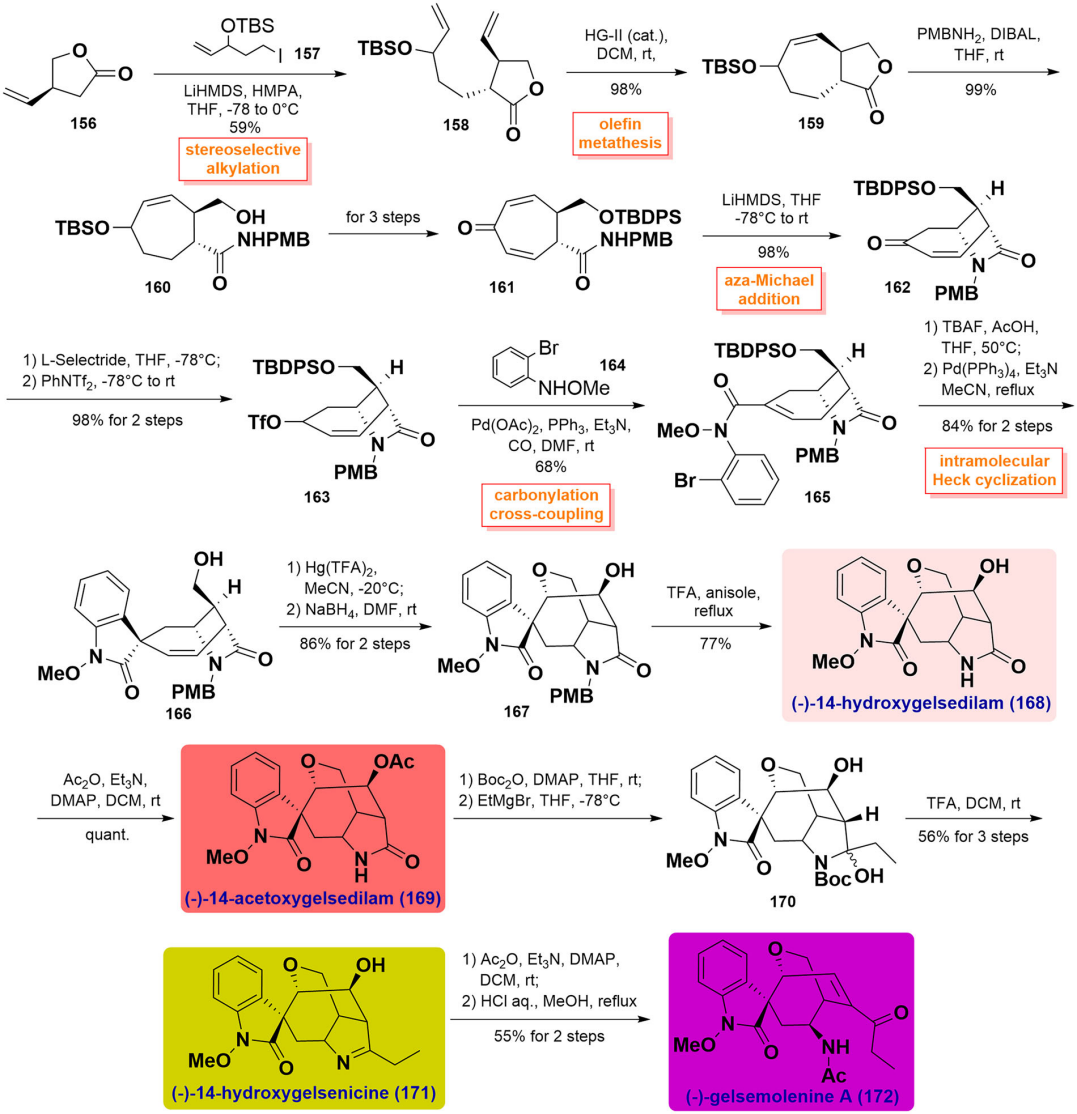

**Figure:**
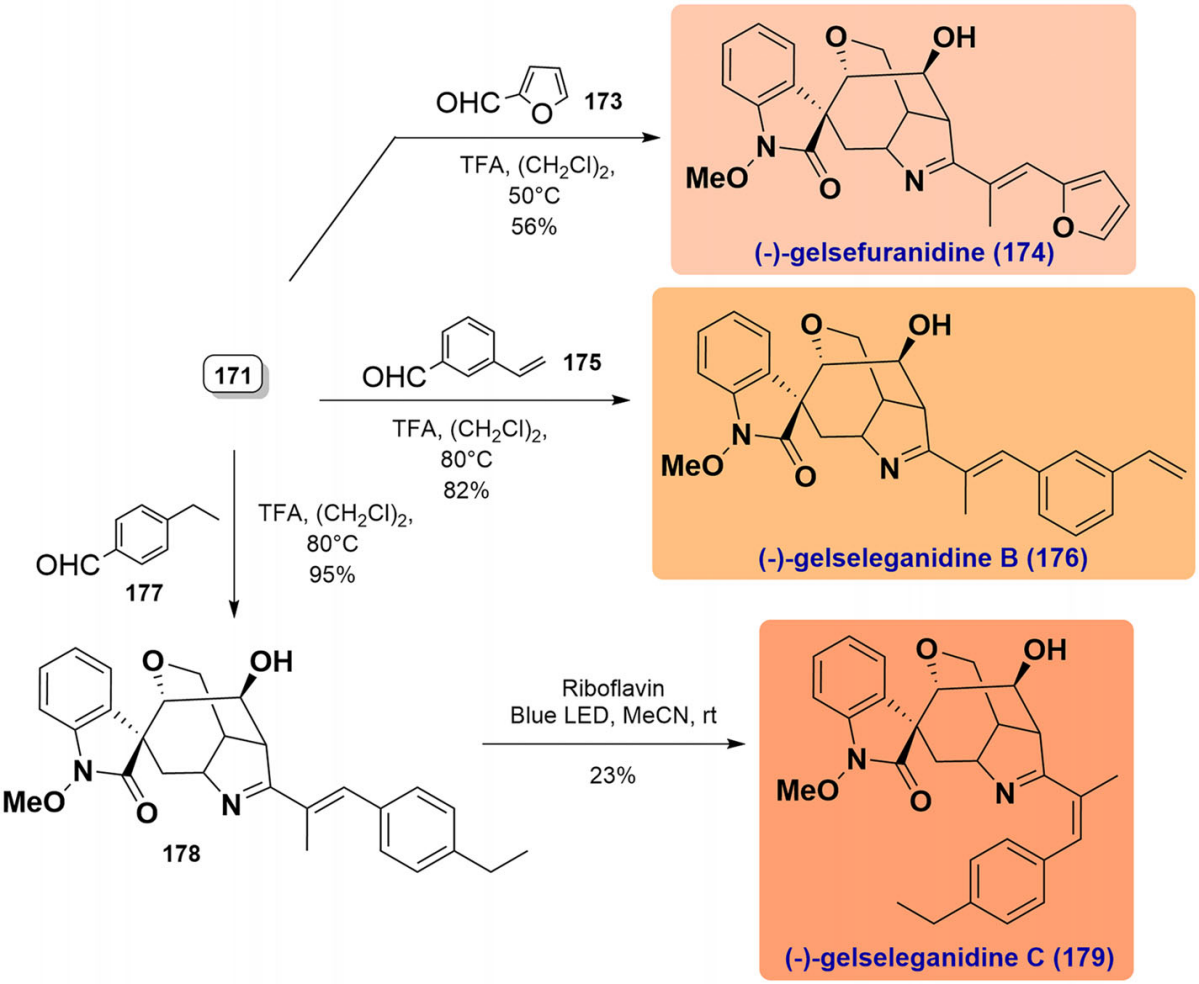

### Total syntheses of gelsemine-type alkaloids

#### Qiu’s total synthesis of (+)-gelsemine (2015)

**Scheme 7:**
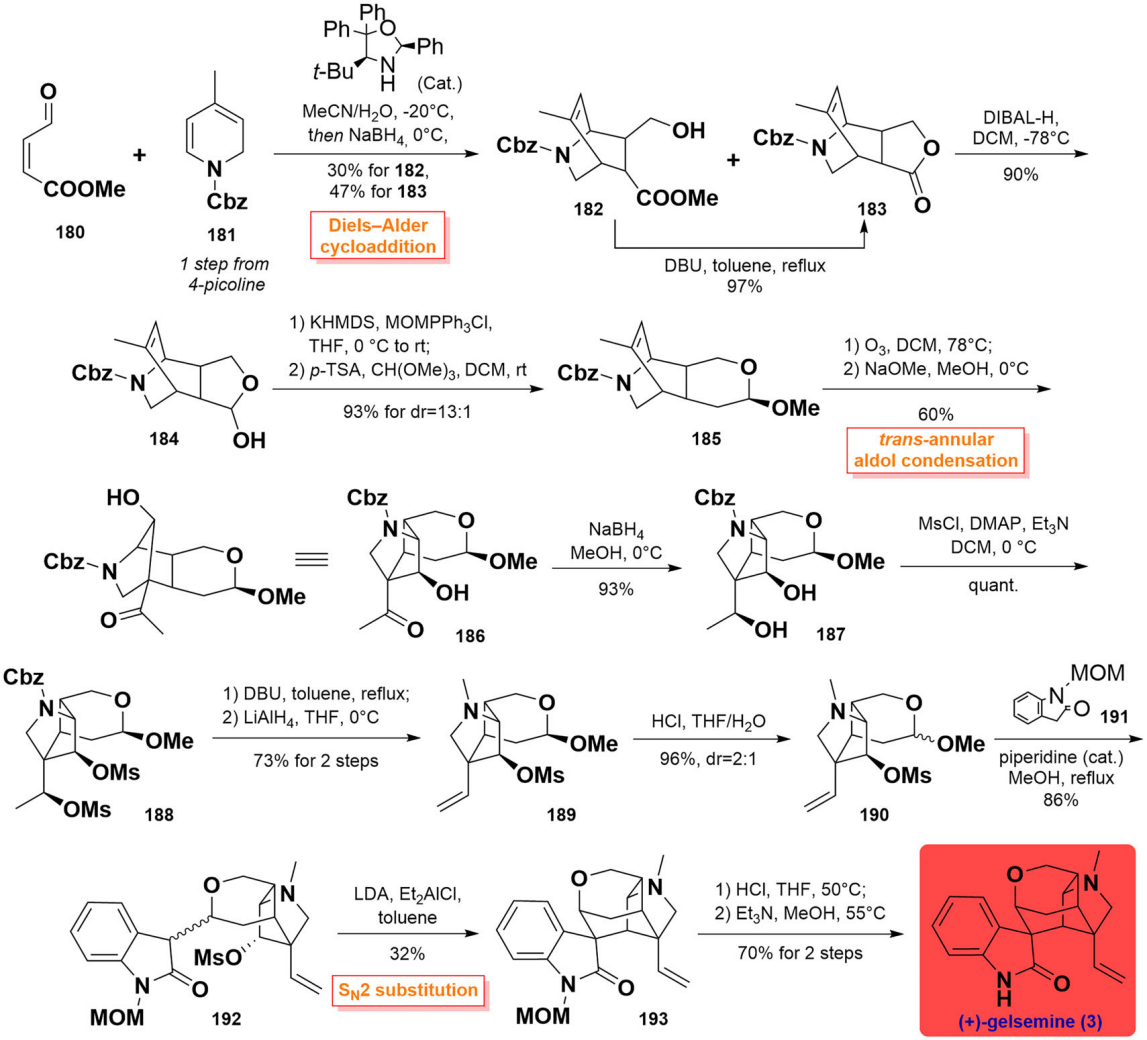
Qiu’s total synthesis of (+)-gelsemine.

In 2015, Qiu and co-workers completed the asymmetric total synthesis of (+)-gelsemine using an organocatalytic Diels-Alder starting strategy^73^. The synthesis was initiated by the linkage of methyl (*Z*)-4-oxobut-2-enoate **180** with dihydropyridine **181** through an organocatalytic Diels-Alder reaction. This process provided the intermediate **183** and by-product **182**. Fortunately, **182** could be converted to lactone **183** through an intramolecular cyclisation in the presence of DBU in 97% yield. **183** was then transformed into hemiacetal **184** through selective reduction using DIBAL-H at −78 °C. It was then subjected to a Wittig reaction to yield a racemic mixture that occurred an electrophilic addition reaction by catalytic *p-*TSA in DCM to afford (*S*)-acetal **185** (dr = 13:1) in 93% overall yield. Subsequent ozonolysis of **185** employing ozone in DCM, accompanied by *trans*-annular aldol condensation of resulting dicarbonyl groups using sodium methanol afforded ketone **186**. On subjecting reduction of **186** using NaBH_4_ led to hydroxyl **187**, whose hydroxyl group was further methanesulfonylated with MsCl to afford disulfonate **188**. Upon treatment of **188** with DBU in heating toluene, reduction of the Cbz protecting group to methyl group with LiAlH_4_ in THF resulted in the formation of olefin **189**. Hemiacetal **190** was prepared via acid hydrolysis with HCl in THF in a 2:1 dr ratio. Then, 1-MOM-oxindole **191** was installed onto **190** via condensation reaction by catalytic piperidine to generate the resulting product **192**. Using their optimised conditions, its treatment with LDA and subsequent S_N_2 substitution reaction using Et_2_AlCl constructed the configuration of the C7 quaternary carbon stereochemical centre and afforded the desired **193** as a single diastereoisomer in only 32% yield. Finally, acid hydrolysis of the methyl group from the MOM group and removal of the resulting hydroxymethyl group using Et_3_N furnished (+)-gelsemine (**3**) in 70% yield over 2 steps. (Scheme 7)

#### Vanderwal’s synthetic route to the polycyclic core of gelsemine (2015)

**Scheme 8:**
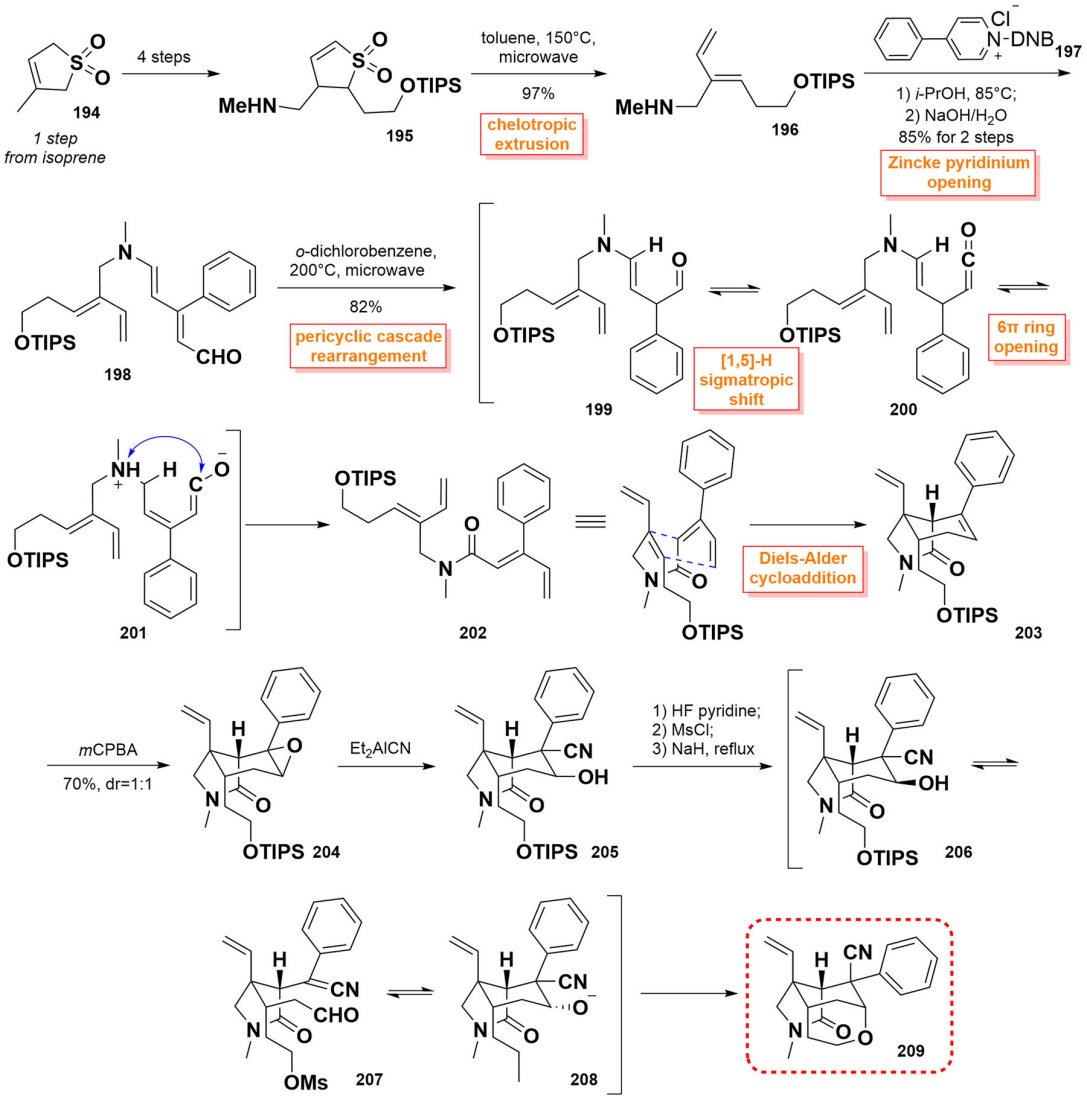
Vanderwal’s synthetic route to the polycyclic core of gelsemine.

In the same year, Vanderwal’s and co-workers relied on a Zincke-aldehyde-based approach to prepare the polycyclic core intermediate to gelsemine^74^. Sulfolene **195** was prepared by the bromination of 3-methylsulfolene **194** (from the adduct of isoprene and SO_2_) through 4 steps. Then, dienyl amine **196** could be accessed through the chelotropic extrusion of SO_2_ under microwave conditions in toluene at 150 °C. Subsequent reaction of **196** with pyridinium salt **197** triggered the pyridine ring-opening, affording Zincke aldehyde **198** in 85% yield. Next, **198** underwent a pericyclic cascade rearrangement including [1,5]-H sigmatropic shift and 6π electrocyclic ring opening to smoothly generate ketone **202** as a single diastereomer under microwave irradiation in 82% yield. With **202** in hand, a concomitant intramolecular Diels-Alder cyclisation was carried out to yield bicyclic lactam **203**, which was prepared to a pair of the separable diastereomeric mixture of epoxide **204** after epoxidation with *m*CPBA. Ring-opening reaction using Nagata’s reagent followed by hydrogenation advanced **204** to the only one secyanoalcohol **205** with excellent diastereoselectivity. It was converted to the critical polycyclic skeleton **209** of gelsemine via a series of reactions involving the switch from TIPS to Ms protecting group, retro-aldol-type cleavage, and pyran ring closure. As the authors’ report, several attempts to partially or fully hydrolyse the nitrile prior to decomposition were not successful. (Scheme 8)

### Total syntheses of koumine and sarpagine-type alkaloids

#### Takayama’s asymmetric total synthesis of koumine (2016)

**Scheme 9:**
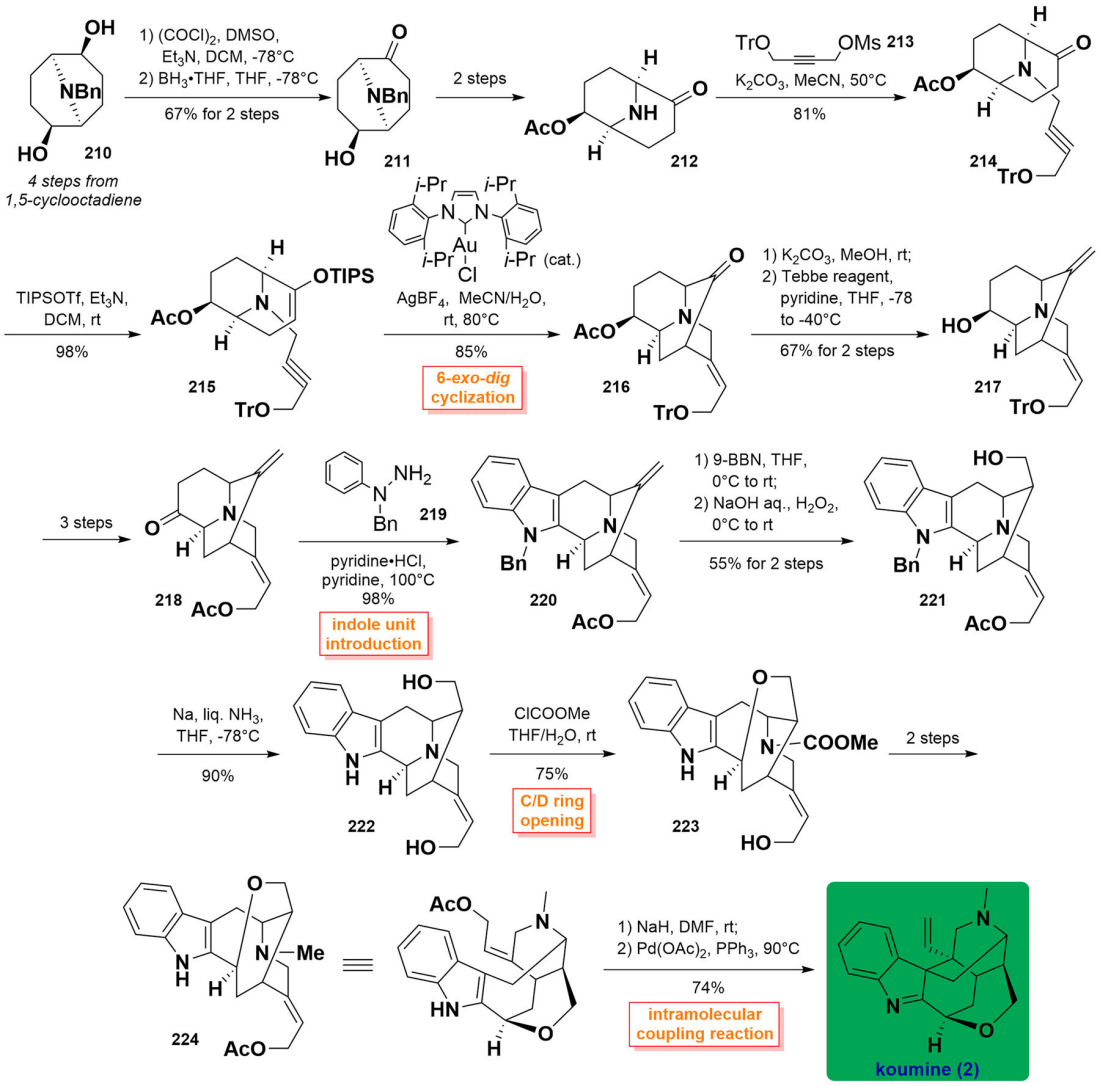
Takayama’s asymmetric total synthesis of koumine.

In 2016, Takayama and co-workers published the asymmetric total synthesis of koumine^75^. Their synthetic strategy relied on a stereoselective gold(I)-catalysed 6-*exo*-*dig* cyclisation reaction providing a key piperidine intermediator with an exocyclic (*E*)-ethylidene side chain. The approach utilised azabicyclononane **210** (4 steps from 1,5-cyclooctadiene) as the starting material, followed by Swern oxidation and secondary selective carbonyl reduction with the use of BH_3_ to generate alcohol **211**. Then, it was transformed into amine **212** through 2 steps. The alkylation reaction of **212** with alkyne **213** resulted in the formation of ether **214**, which was then converted into silyl enol ether ester **215**. Gold(I)-catalysed 6-*exo*-*dig* cyclisation of **215** using Au catalysis/AgBF_4_ in MeCN/H_2_O at 80 °C furnished the common intermediate **216** in 85% yield. Deprotection of the acetyl group allowed the free hydroxyl group along with the conversion of a ketone into olefin on treating Tebbe reagent to form olefinic **217**, which proceeded to yield ketone **218** after 3 steps. The reaction of **218** with phenylhydrazine **219** in the presence of pyridine·HCl initiated the construction of an indole unit to form *N*-benzyl indole **220** in 98% yield. Successively, a 9-BBN-induced borohydride reaction followed by oxidation with H_2_O_2_ was achieved to regio- and diastereoselectively introduce a hydroxyl group at C17 in **221**. **221** was subjected to *N*-benzyl deprotection using Na/liquid NH_3_ in THF rendering the formation of indole **222**. After the successful preparation of indole **222**, Takayama and co-workers turned our attention to the synthesis of koumine. The C/D ring opening was achieved upon treatment with methyl chloroformate in THF/H_2_O to afford the resulting intermediate **223**, which was transformed to **224** over 2 steps. Finally, NaH-treated indole ionisation followed by Pb(OAc)_2_-catalysed intermolecular indolyl addition to the allene chain built the C20-C7 bond, thus furnishing the desired koumine (**2**). (Scheme 9)

#### Kerr’s total synthesis of isodihydrokoumine and (4 R)-isodihydroukoumine N_4_-oxide (2018)

**Scheme 10:**
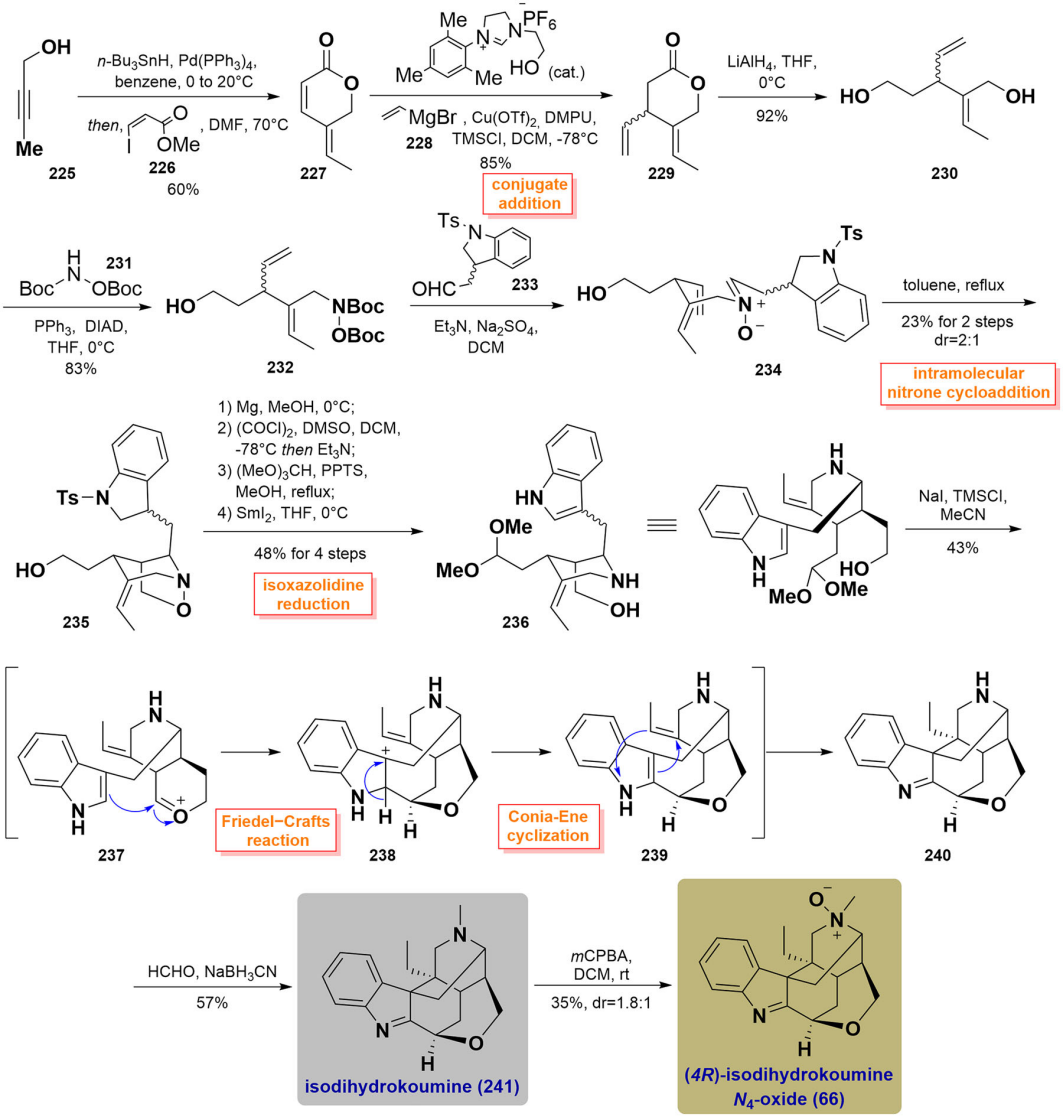
Kerr’s total synthesis of two koumine-type alkaloids.

In 2018, Kerr’s total syntheses of isodihydrokoumine and (4 *R*)-isodihydroukoumine *N*_4_-oxide utilised an intramolecular [3 + 2] nitrone olefin cycloaddition and a Lewis acid-mediated cyclisation as the key steps to prepare their core structure^76^. They commenced this synthetic study with the preparation of dihydropyranone **227**. Hydrostannylation of alkyne **225** with *n*-Bu_3_SnH followed by Stille coupling with methyl (*Z*)-3-iodoacrylate **226** provided dihydropyranone **227** in 60% yield. Then, the copper-catalysed conjugate addition of **228** with vinyl magnesium bromide **228** led to the formation of lactone **229**. After the smooth reduction of lactone, the allylic alcohol of the resulting product was substituted through Mitsunobu reaction to yield hydroxylamine **232** in 83% yield with high regioselectivity. Upon removal of the Boc groups, the relevant product was condensed with *N*-tosyl indole-3-acetaldehyde **233** *in situ* to provide nitrone **234**, which underwent an intramolecular *N*-alkenyl nitrone dipolar cycloaddition upon heating in toluene to produce isoxazolidine **235** in a 23% yield of a 2:1 mixture of *cis-*diastereomer. Removal of the *N*-tosyl protecting group with Mg, followed by Swern oxidation, acetal protection as well as SmI_2_-mediated isoxazolidine reduction, and ring-opening smoothly delivered acetal **236** in 48% yield over 4 steps. Treatment of **236** with TMSCI in MeCN induced the cascade Friedel-Crafts and Conia-Ene cyclizations that forged the polycyclic cage skeleton in **240**. Eschweiler-Clarke reaction installed a methyl group on an N4 atom and then furnished natural isodihydrokoumine (**241**) in a 57% yield. Oxidisation of the N4 atom in **241** with *m*CPBA generated the separable diastereomeric products in a dr ratio of 1.8:1, which was separated by chiral chromatography to afford the natural product (*4 R*)-isodihydrokoumine-*N*_4_-oxide (**66**) in a 35% yield. (Scheme 10)

#### De Paolis’ asymmetric synthetic route to the bicyclic core of koumine (2019)

**Scheme 11:**
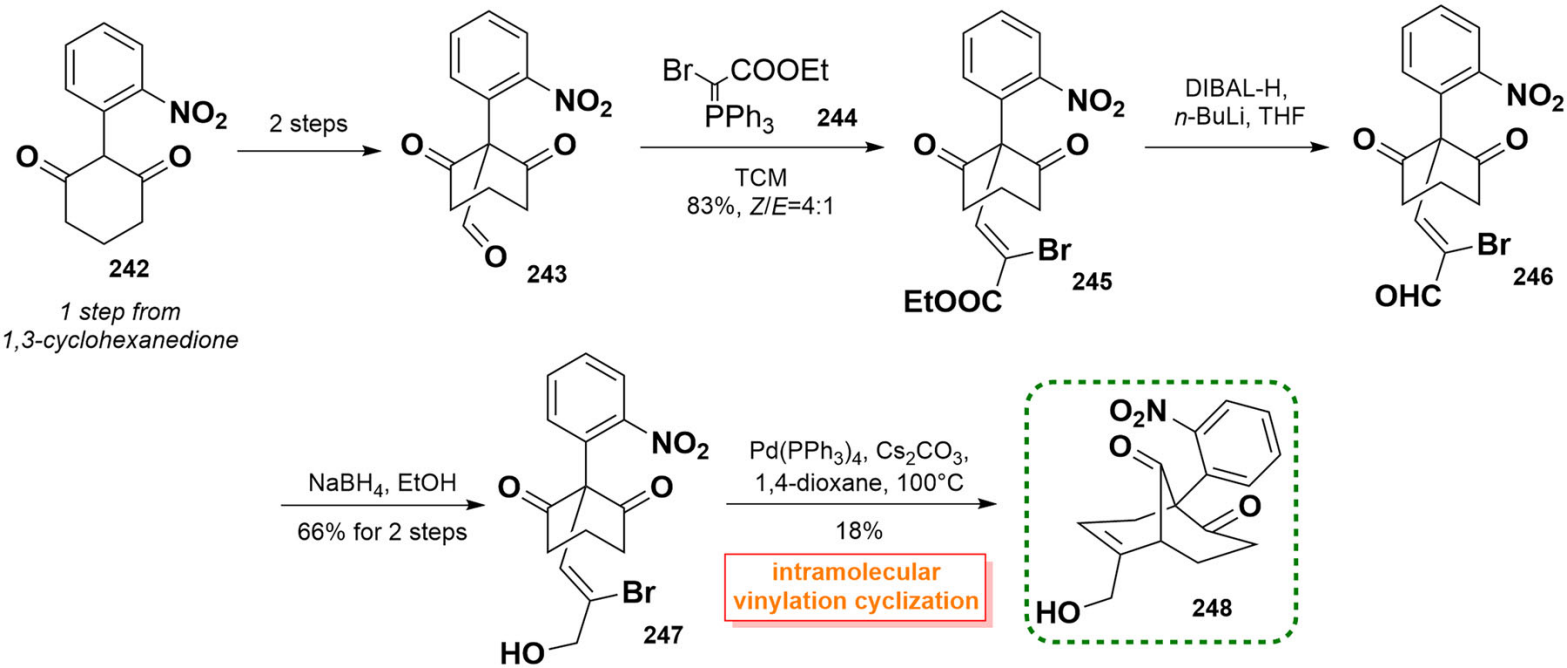
De Paolis’ asymmetric synthetic route to the bicyclic core of koumine.

In 2019, De Paolis and co-workers utilised an intramolecular vinylation reaction of an enolate to form a [3.3.1] bicyclononane framework^77^. In their synthetic pathway, the aldehyde **243** was yielded from 2-nitrophenyl-1,3-cyclohexanedione **242** (1 step from 1,3-cyclohexanedione) through 2 steps. Then, **243** was condensed with ylide **244** through Wittig reaction to afford ester **245** in 83% yield with a high level of *Z*/*E* selectivity (4:1). Subsequent treatment of DIBAL-H/*n*-BuLi followed by reduction with NaBH_4_ in THF caused the construction of vinyl bromide **247**. Intramolecular vinylation cyclisation using catalytic Pd(PPh_3_)_4_ initiated the formation of the crucial [3.3.1] bicyclononane core, thus giving the expected intermediator **248**, albeit in 18% yield. With **248** in hand, there was an opportunity to employ this key core as the progenitor needed for the synthesis of koumine (**2**). It was envisioned that koumine eventually could be completed by subsequent closure of the piperidine and tetrahydropyrane ring as well as the assembly of the indole portion. (Scheme 11)

#### Tanja’s total synthesis of koumidine (2019)

**Scheme 12:**
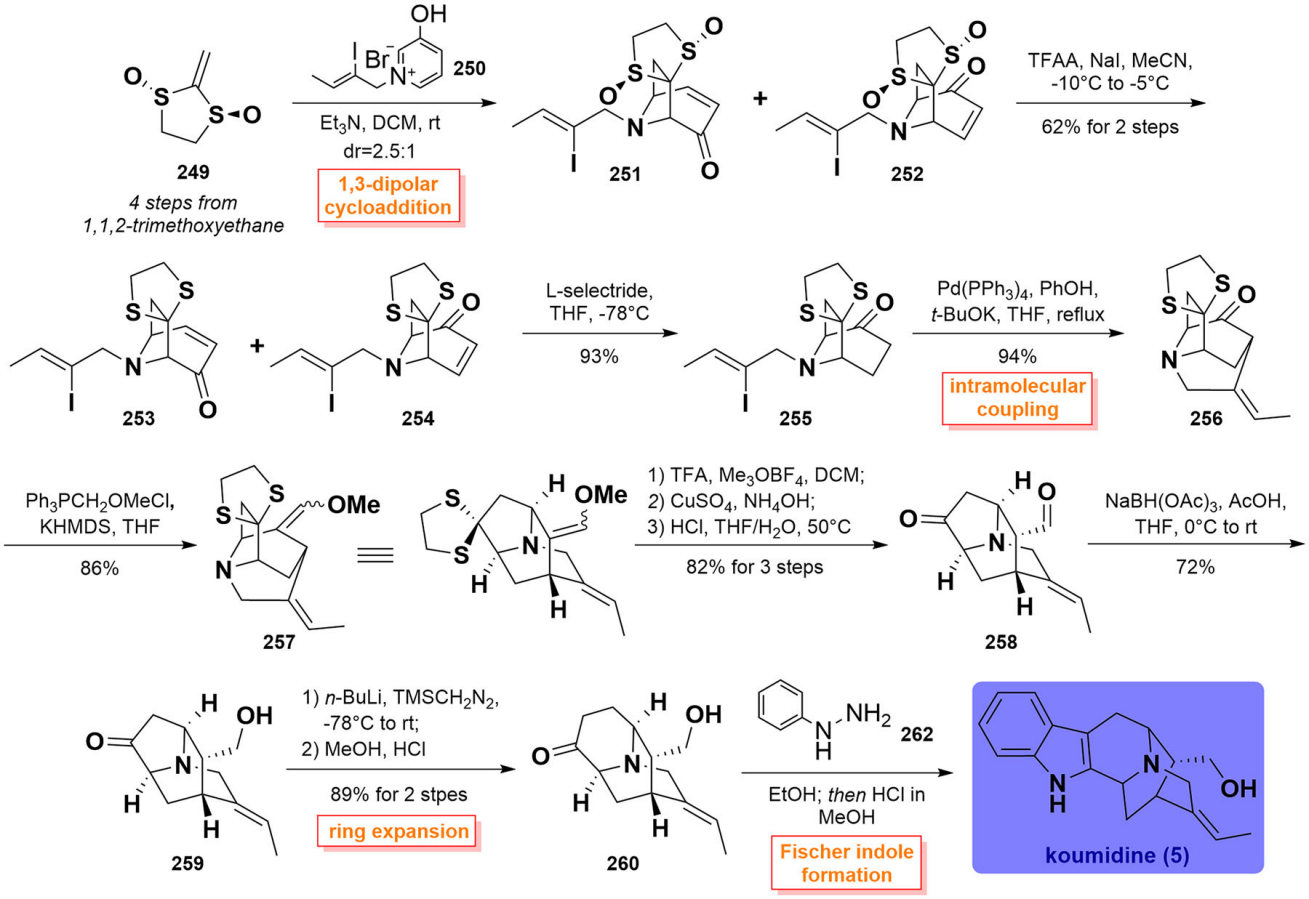
Tanja’s total synthesis of koumidine.

In Tanja’s synthesis of koumidine, the late-stage enol-oxonium cyclisation sequence was used to construct the hexacyclic cage framework on a gram scale^78^. In their work, a highly diastereoselective 1,3-dipolar cycloaddition reaction of *trans*-2-methylene-1,3-dithiolane 1,3-dioxide **249** (from available 1,1,2-trimethoxyethane in 4 steps) with 3-oxidopyridinium **250** generated an inseparable mixture of tropane **251** and **252** in 2.5:1 dr ratio. Next, the bissulfoxides in the mixture of **251** and **252** were simultaneously reduced using TFAA/NaI to afford two separable regioisomers dithiolanes **253** and **254**, with the latter capable of being transformed to ketone **255** via 1,4-reduction using L-selectride. Then, the Palladium-catalysed intramolecular coupling of vinyliodide and ketone fused the piperidine ring using potassium phenoxide in THF, thus affording tetracycle **256**. Wittig reaction of **256** with triphenylmethylmethoxy-chloride in the presence of KHMDS happened to smoothly deliver the corresponding enol ether **257** with high efficiency. After removal of the ethylenethioacetal group using Meerwein’s reagent, the resulting bissulfonium intermediate was hydrolysed with CuSO_4_, followed by basification with NH_4_OH to yield the crude mixture, which was readily converted to keto-aldehyde **258** via acid hydrolysis in 82% yield over 3 steps. Subsequently, the chemoselective reduction of the aldehyde proceeded to yield alcohol **259** with excellent chemoselectivity that participated in a TMSCH_2_N_2_-involved expansion reaction followed by subsequent resulting TMS enol ether hydrolysis to furnish 6-membered ketone **260.** With sufficient **260** in hand, the total synthesis of koumidine (**5**) was eventually completed by Fischer indole synthesis reaction with phenylhydrazine **261**. (Scheme 12)

#### Zhang’s asymmetric total syntheses of (-)-koumimine, (-)-N-demethylkoumine, and (-)-koumine (2021)

**Scheme 13:**
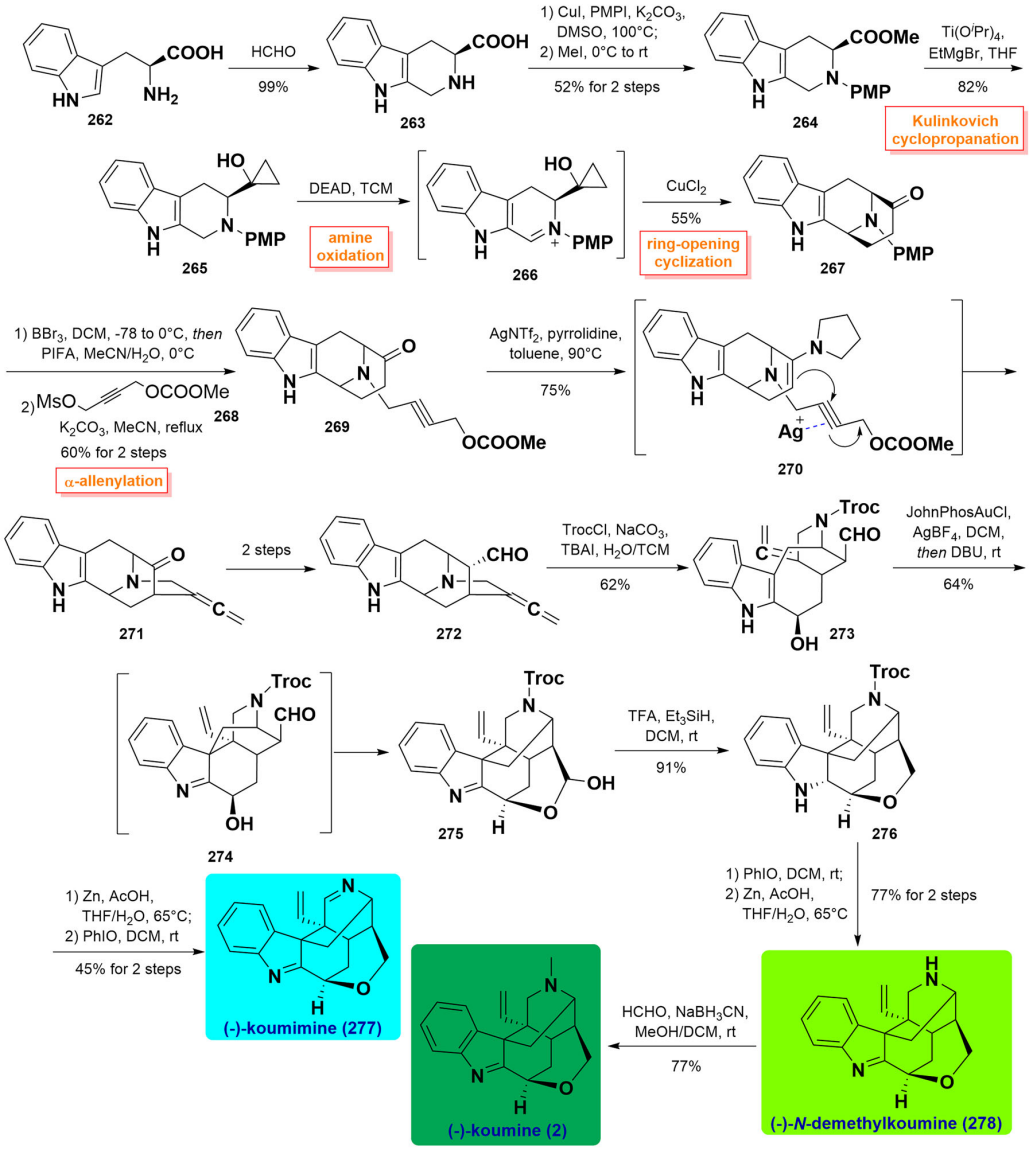
Zhang’s total syntheses of three koumine-type alkaloids.

In 2021, Zhang and co-workers disclosed a tandem sequential oxidative cyclopropanol ring-opening cyclisation and a cooperative organo/metal-assisted ketone α-allenylation for constructing the core skeleton of koumine^79^. Methyl ester **264** was first prepared from L-tryptophan **262** in 3 steps involving by Pictet-Spengler reaction, *N*-arylation, and carboxylic acid esterification. **264** underwent Kulinkovich cyclopropanation of the carboxyl ester to furnish cyclopropanol **265**, which was directly subjected to DEAD-promoted amine oxidation and subsequent CuCl_2_-catalysed cyclopropanol ring-opening cyclisation to set up the bicyclo[3.3.1]nonane in **267**. Dearylation of **267** and secondary alkylation of the free amine with **268** afforded **269** in 60% yield for 2 steps. Under identical reaction conditions, treatment of **269** with pyrrolidine and AgNTf_2_ facilitated the formation of the bicyclo[2.2.2]octane to afford ketone **271** in 75% yield. Then, it could be converted to aldehyde **272** in 2 steps. Its treatment with TrocCl and an excess Na_2_CO_3_ led to alcohol **273** as a single diastereomer in 62% yield. The synthesis was further manipulated by a Gold-catalysed intramolecular coupling reaction between C7 and C20 positions, DBU-induced isomerisation of the aldehyde group, and sequential aldol condensation reaction with trapping of the hydroxyl group newly generated *in situ* to obtain the required hemiacetal **275** as a single diastereomer. Next, the hemiacetal and imine moieties were reduced by TFA/Et_3_SiH to produce the resulting **276**. Removal of the Troc group and successive PhIO-induced oxidation gave (-)-koumimine (**277**) in 45% yield over 2 steps. Meanwhile, PhIO-exposed oxidation and subsequent deprotection of the Troc group took place to complete the synthesis of (-)-*N*-demethylkoumine (**278**) in 77% yield. Finally, a HCHO/NaBH_3_CN-assisted reductive methylation happened to afford (-)-koumine (**2**) in good yield. (Scheme 13)

#### Zhang’s total syntheses of akuammidine, 19-(Z)-akuammidine, komidine, dihydrokoumine and koumine (2022)

In 2022, Zhang and co-workers exploited a unified approach towards the asymmetric synthesis of sarpagine- and koumine-type alkaloids. Among them, akuammidine, 19-*Z*-akuammidine, and dihydrokoumine are synthesised for the first time^80^. The synthesis started with the preparation of sulfinamide **283** through Schiff base formation and a vinylogous Mannich reaction from (*3S*)-aldehyde **279** (from tryptophol for 2 steps) in 72% yield. Then, **283** was converted to unstable aldehyde **284** over 4 steps. The azabicyclo[3.3.1]nonane in **285** could be built through an intermolecular cyclisation in the presence of Lewis acid, which was further transformed to **286** over 3 steps. Alkylation of olefin with 3–(2-bromoacetyl)oxazolidin-2-one **287** was carried out to afford *N*-acyl-oxazolidinone **288**. Next, SmI_2_-mediated asymmetric radical cyclisation smoothly proceeded to fuse the bridged piperidine-3-one ring, thus affording ketone **291** with excellent diastereoselectivity. Its olefination reaction with Julia reagent **292** exhibited preference for (*Z*)-olefin **293** as the common sarpagine-type skeleton in 80% yield with a dr ratio of 4.2:1. Treatment of **291** with Wittig reagent in THF in the presence of NaHMDS provided (*E*)-olefin **294** in 83% yield with a dr ratio of 5.5:1. Then, the first total synthesis of akuammidine (**295**) and 19-(*Z*)-akuammidine (**296**) were completed from **293** and **294** via Knoevenagel condensation reaction with formaldehyde followed by deprotection of the PMB group, respectively.

On the other hand, treatment of intermediate **294** with I_2_ in the presence of LDA yielded iodide **297**. The required stereochemistry at C16 in **298** was fixed by light-induced radical reduction of **297** with catalytic [Ir(ppy)_2_(dtbbpy)]PF_6_ with the use of DIPEA and (TMS)_2_NH under blue LED at −60 °C in 47% yield. Koumidine (**5**) could be obtained from the removal of the PMB group of **298** with TFA along with reduction with LiAlH_4_ in 80% yield over 2 steps. Treatment of **5** with methyl chloroformate triggered the formation of amide **299** in a 69% isolated yield. NIS-induced cyclisation simultaneously set up two vicinal all-carbon quaternary stereocenters, thereby providing iodide **300** in 88% yield. Next, olefin **301** was obtained by eliminating the iodide in **300** with AgOAc in acetic acid. Upon reduction with LiAlH_4_, the first synthesis of dihydrokoumine (**302**) was eventually completed in 79% yield. Oxidation of **302** with PhIO in DCM gave rise to koumine (**2**) in nearly quantitative yield. (Scheme 14)

Scheme 14Zhang’s total syntheses of sarpagine- and koumine-type alkaloids.

**Figure:**
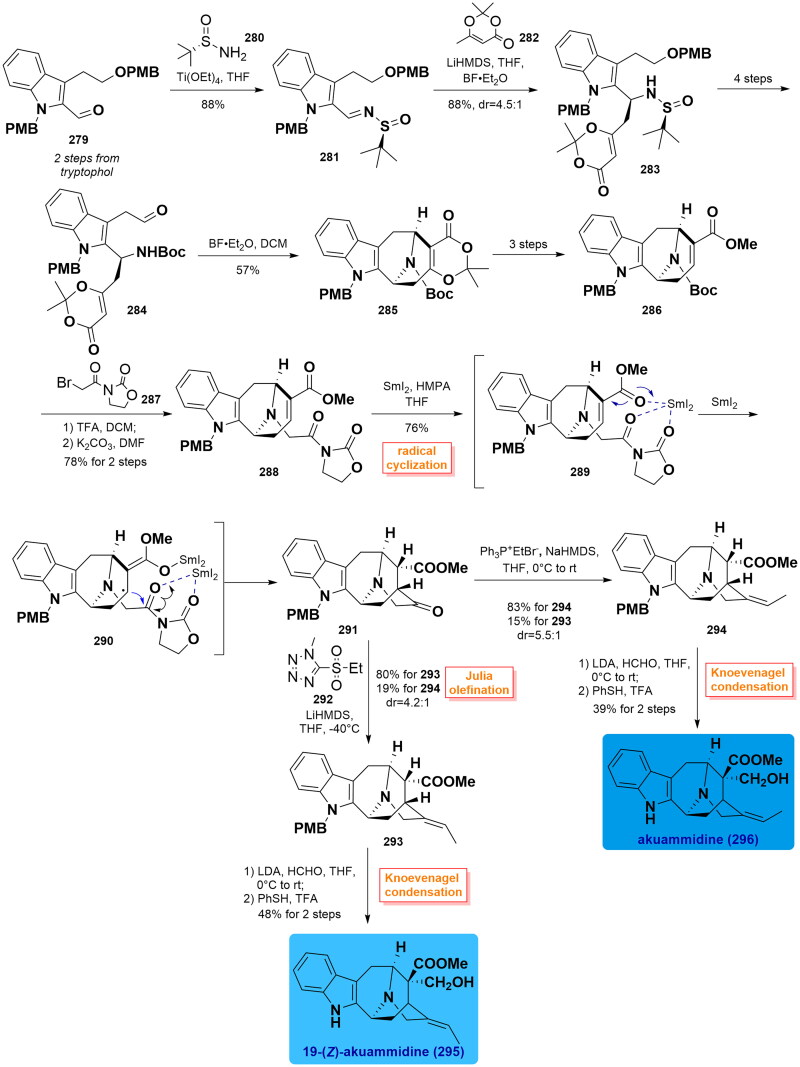

**Figure:**
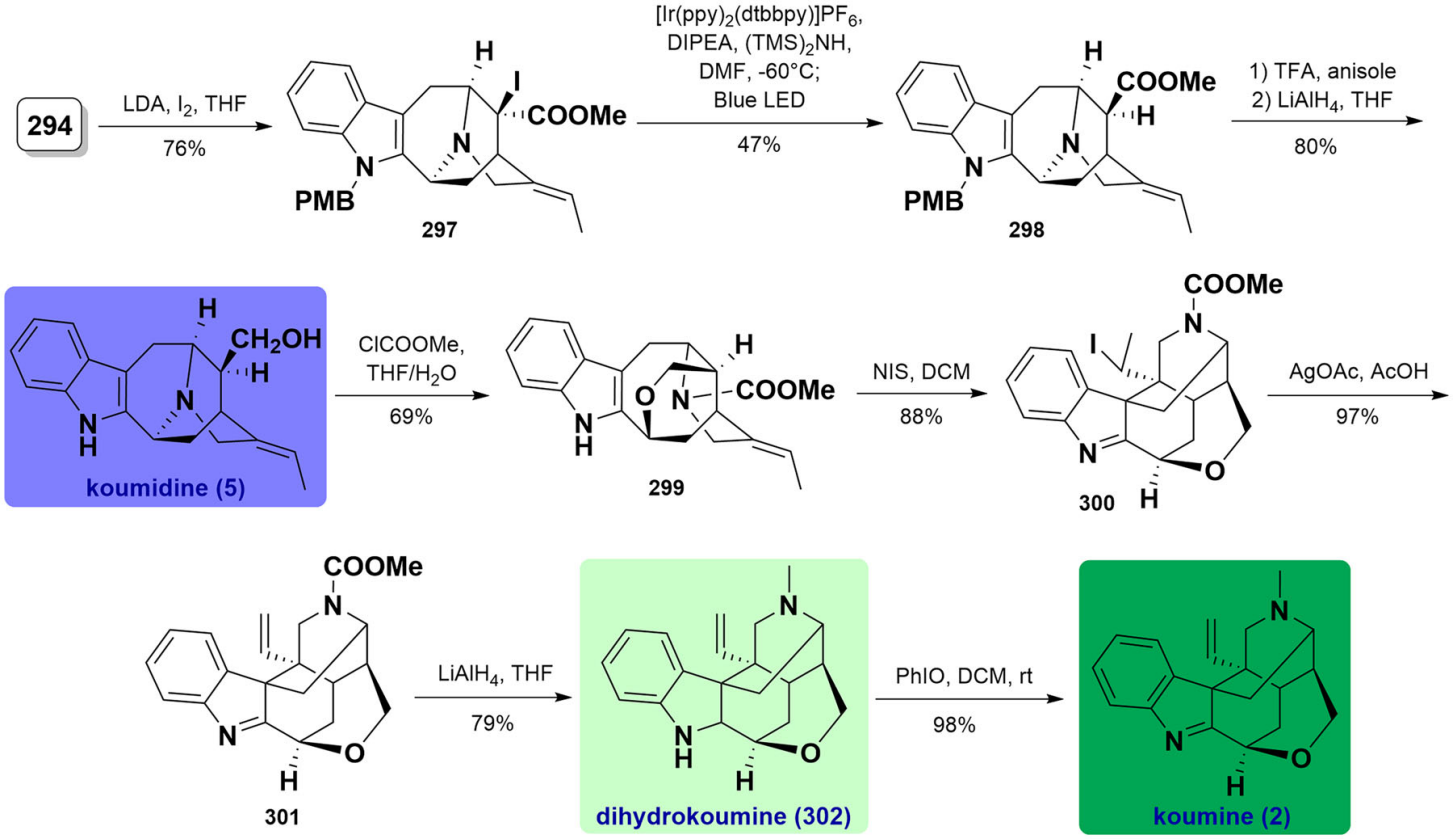

### Total syntheses of sempervirine-type alkaloids

#### Malhotra’s total synthesis of sempervirine (2013)

**Scheme 15:**
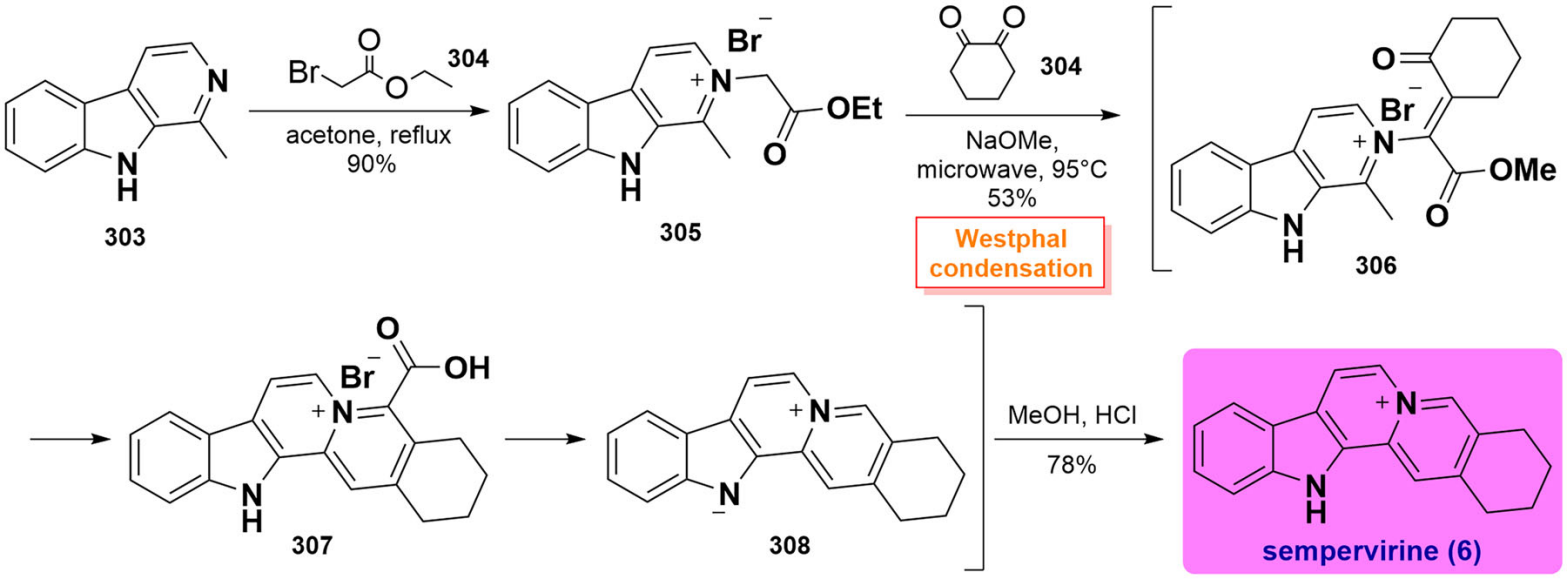
Malhotra’s total synthesis of sempervirine.

In 2013, Malhotra and co-workers accomplished the concise total synthesis of sempervirine under microwave irradiation in one pot process^81^. Quaternization of 1-methyl-9*H*-pyrido[3,4-b]indole **303** with ethyl bromoacetate **304** occurred to give the corresponding quaternary **305** in excellent yield. In a one-pot reaction, a Westphal condensation reaction with 1,2-cyclohexanedione **306** under microwave heating in MeOH in the presence of sodium methoxide, followed by ester hydrolysis and decarboxylation yielded a separable mixture of sempervirine precursor **309** in 53% yield. After acidification with dilute hydrochloric acid, the mixture was separated through silica gel chromatography to afford the expected sempervirine (**6**) in a 78% isolated yield. (Scheme 15)

#### Bannister’s total synthesis of sempervirine triflate (2016)

**Scheme 16:**
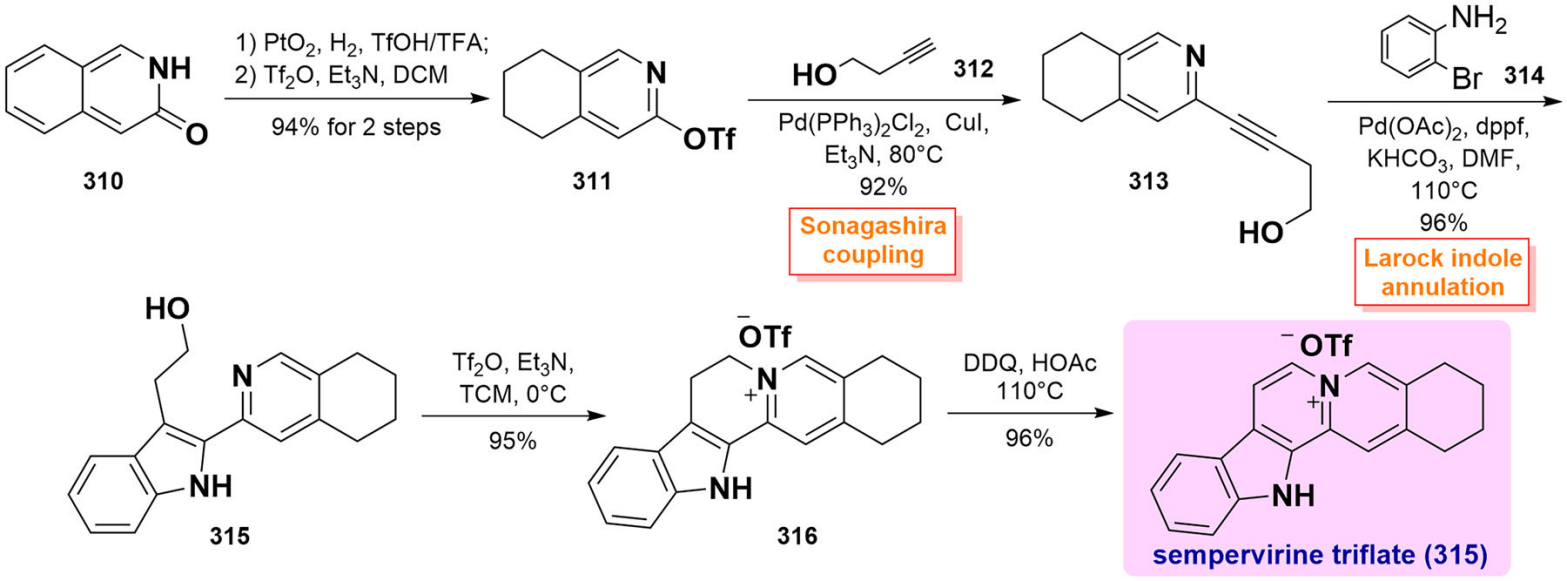
Bannister’s total synthesis of sempervirine triflate.

In 2016, Bannister and co-workers used Pd-catalysed Sonagashira coupling and Larock indole annulation reaction to efficiently synthesise sempervirine and its analogs^82^. Regioselective semi-reduction of 3-isoquinolone **310** using PtO_2_ catalysis in TfOH/TFA generated the corresponding hydrogenated product, whose amide group was further alkylated to provide triflate **311** in 94% yield over 2 steps. Under optimised Sonagashira conditions, the addition of **311** to butyne-1-ol **312** smoothly gave alkyne **311** using Pd(PPh_3_)_2_Cl_2_ as a catalytic agent in 92% yield. Upon Larock indole synthesis of **313** with *o*-bromoaniline **314** catalysed by Pd(OAc)_2_, the 2-heteroaryl indole product **315** was prepared with a good yield. Subsequently, a triflate-mediated cyclisation cleanly furnished the intermediate pyridinium salt **316**. Ultimately, it was converted to sempervirine triflate (**317**) through DDQ-promoted oxidation in 96% yield. (Scheme 16)

## Conclusions and perspectives

At present, this review has summarised a total of 70 novel gelsemium MIAs, which greatly complemented the library of compounds from the *Gelsemium* genus. Although remarkable accomplishments to synthesise several gelsemium MIAs are successful, the separation from plants are a more economical route for access to these gelsemium MIAs and their analog, due to their abundance in plant sources. An increasing number of gelsemium MIAs are essential for mapping out their possible biosynthesis mechanisms and molecule transformations. Once a reasonable biosynthesis is proposed, it is conducive to satisfying the need of synthetic chemists for addressing the synthetic challenges of complex gelsemium MIAs depending on biomimetic synthesis inspired by nature. Therefore, it is necessary to deeply dig and identify unknown trace gelsemium MIAs from natural sources on a large scale. It is noteworthy that the structure-activity relationship of many gelsemium MIAs remains unclear, partly because of limited material availability. The structural modification of gelsemium MIAs using semi-synthesis directly starting from several key intermediates could effectively solve this issue as possible. Besides, these synthetic tactics also enable to provide novel gelsemium MIAs derivatives with better bioactivities. Further innovative total synthetic strategies with more concise steps and higher yields should be designed to construct the complicated skeletons of gelsemium MIAs.

Despite gelsemium MIAs’ promising bioactive potentials both *in vitro* and *in vivo*, their exact molecular mechanisms and specific targets in many types of diseases have long been limited; thus, such further studies are required. Bioinformatics and the integration analyses of transcriptome, genome, intestinal flora, and proteome are ripe for wide prediction and exploration of singling pathways and precise target proteins^83–85^. Molecular docking tools, which create ligand-target interaction, aid the prediction of binding sites of compounds and the delineation of SARs^86^. These approaches greatly support the in-depth understanding of gelsemium MIAs-regulated molecular mechanisms. In addition, the extensive clinical applications of gelsemium MIAs and plants are still largely challenging due to their toxicity. Our study has reported that the combination treatment of koumine and *Glycyrrhiza uralensis* showed a significant low-toxic effect by upregulating cytochrome enzymes and mediating pharmacokinetics^87^. Thus, the synergistic application with another detoxifying agent would be crucial to partly enhancing the curative effect and reducing the toxicity of individual gelsemium MIA or gelsemium extract. Regarding the tissue distribution, the koumine and gelsemine peak concentrations in the intestines and livers are higher than that in other tissues, thus indicating that gelsemium MIAs may bring greater advantages into full play in the treatment of digestive system diseases^88^^,^^89^.

In conclusion, a historical account of relevant studies research advances on the structural diversity, potential bioactivity, and total syntheses of gelsemium MIAs covering the period from 2013 to 2022 has been described and discussed. We hope this review will help drive the future drug development of gelsemium MIAs as promising lead compounds with better safety and potency forward.
